# Biases in estimation of insect herbivory from herbarium specimens

**DOI:** 10.1038/s41598-020-69195-5

**Published:** 2020-07-23

**Authors:** Mikhail V. Kozlov, Irina V. Sokolova, Vitali Zverev, Alexander A. Egorov, Mikhail Y. Goncharov, Elena L. Zvereva

**Affiliations:** 1 Department of Biology, University of Turku, 20014 Turku, Finland; 2 Herbarium, V. L. Komarov Botanical Institute, Professora Popova Str. 2, 197376 St. Petersburg, Russia; 3 Department of Biogeography and Nature Preservation, Institute of Earth Sciences, St. Petersburg State University, Universitetskaya nab. 7-9, 199034 St. Petersburg, Russia; 4 St. Petersburg Chemical-Pharmaceutical University, Professora Popova Str. 14, 197022 St. Petersburg, Russia

**Keywords:** Ecology, Plant sciences

## Abstract

Information regarding plant damage by insects in the past is essential to explore impacts of climate change on herbivory. We asked whether insect herbivory measured from herbarium specimens reflects the levels of herbivory occurring in nature at the time of herbarium sampling. We compared herbivory measurements between herbarium specimens collected by botany students and ecological samples collected simultaneously by the authors by a method that minimized unconscious biases, and asked herbarium curators to select one of two plant specimens, which differed in leaf damage, for their collections. Both collectors and curators generally preferred specimens with lesser leaf damage, but the strength of this preference varied among persons. In addition, the differences in measured leaf damage between ecological samples and herbarium specimens varied among plant species and increased with the increase in field herbivory. Consequently, leaf damage in herbarium specimens did not correlate with the actual level of herbivory. We conclude that studies of herbarium specimens produce biased information on past levels of herbivory, because leaf damage measured from herbarium specimens not only underestimates field herbivory, but it is not proportional to the level of damage occurring in nature due to multiple factors that cannot be controlled in data analysis.

## Introduction

The data on the intensity of biotic interactions during pre-industrial times are badly needed for evaluation of the extent of the pervasive influence of human-induced global environmental changes on organisms, populations, communities and entire ecosystems. Nevertheless, these data are in short supply^1^, particularly for the analysis of insect herbivory, which affects the productivity of ecosystems, modifies nutrient cycling and maintains the diversity of plant communities^2–4^. Exploration of past changes in insect herbivory is crucial for understanding current trends and making justified predictions about the future impacts of insect herbivory on ecosystems, but the earliest quantitative information on losses of woody plant foliage to insects (i.e. on the levels of insect herbivory in natural forest ecosystems) goes back only to 1952^5^. Not surprisingly, ecologists are constantly looking for data sources that can be used to study long-term trends in plant damage by insects. To date, tree growth data obtained from the widths of annual rings is one of the best indices used thus far to explore herbivory at a millennial scale^6^^,^^7^. However, tree growth records only reflect severe, outbreak-level damage and are unsuitable for studies of background insect herbivory.

Natural history collections are a rich source of ecological and environmental information. In particular, herbaria were recently advertised as reliable data resources for the study of historical changes in insect herbivory and in plant damage by pathogens^8–10^. However, sampling and measurements procedures associated with evaluations of herbivory are prone to numerous biases, both conscious and unconscious^11^^,^^12^, which can lead to inaccurate conclusions about the levels of herbivory occurring in nature. Therefore, the use of herbarium specimens for quantitative evaluation of plant losses to insects requires careful exploration of biases that may occur in these types of studies. Much evidence indicates that these biases are likely to exist. For example, botanists have always been advised to collect specimens that bear no or few signs of damage: “specimens… should be in a perfect state of growth, their leaves and other parts uninjured”^13^; “the least injured plants should be chosen, and leaves with margin and apex entire”^14^; “if possible, they [specimens] should be free from the ravages of insects and diseases”^15^; “plants damaged by insects or browsed by mammals, as a rule, are not suitable for sampling”^16^; “specimens are made from freshly cut and undamaged plants”^17^.

The biases in plant sampling for museum collections, and in their subsequent accession and de-accession, are well known to botanists^18^. Analysis of approximately 5 million herbarium records identified multiple spatial, temporal, trait, phylogenetic and collector biases^19^. Another study revealed strong collecting biases against introduced plants, plants with green or brown inflorescences, and very small plants^20^. Some ecological studies have demonstrated the possibility of accounting for biases associated with the use of herbarium collections while reconstructing ecological patterns^21,22^. At the same time, the authors of recent papers that used herbarium specimens to estimate trends in insect herbivory^9,10,23,24^, even though they acknowledge the existence of biases, do not provide any quantitative estimates that would account for the impacts of these biases on their herbivory data.

Both ecologists and entomologists have long been aware of the value of herbaria, and many have used herbarium specimens in their research to address herbivory. Early uses of herbarium specimens generally concerned recording of identifiable insect damage (e.g. leaf mines), and the documentation of this damage in herbarium specimens has contributed to the analysis of past and recent distribution patterns of these insects^25–28^. One of the first attempts to use herbarium specimens for measurements of plant losses to insects was published three decades ago^29^, but subsequent publications utilising the same approach were virtually absent until the 2010s^18^. This absence, in our opinion, indicates that scientists working on insect herbivory understand the problems associated with the use of herbarium specimens and have refrained from their use as a data source for the reconstruction of spatial and temporal patterns in plant losses to insects (with a few justified exceptions^26^).

The goal of the present study was to test the hypotheses that (1) the analysis of herbarium specimens generates biased data on losses of plant foliage to insects, but (2) these biases can be corrected based on cross-calibration procedures. We experimentally tested whether foliar damage recorded from herbarium specimens reflects the actual levels of herbivory at a study site at the time of herbarium sampling, and we asked (1) whether the collectors and curators of botanical collections, as well as other scientists working in herbaria, showed preferences/avoidances of plants with respect to their damage by insects; (2) whether the strength of these preferences/avoidances varies among individual collectors and curators, and (3) whether this preference/avoidance varies with plant species and with the absolute levels of plant damage caused by insects.

## Results

### Effect of sampling protocol on leaf area lost to insects

We analysed 248 samples collected from 17 species of woody plants native to the study region. Among these, 85 samples were collected using the protocol developed for ecological research, and 163 samples were collected as herbarium specimens. Each herbarium specimen, on average, contained four-fold fewer leaves than a sample collected by ecological methods (13.5 and 50.3 leaves, respectively).

Measurements of leaf area lost to insects were performed by M.V.K., who was aware of research hypothesis and sample origin, and by J. Rikus, who was blinded to these factors. The measurements by both persons yielded the same results (average difference ± SE: 0.52 ± 0.48%; *P* > 0.38), indicating that the results by M.V.K. used in subsequent analyses were not affected by confirmation bias.

**Figure 1:**
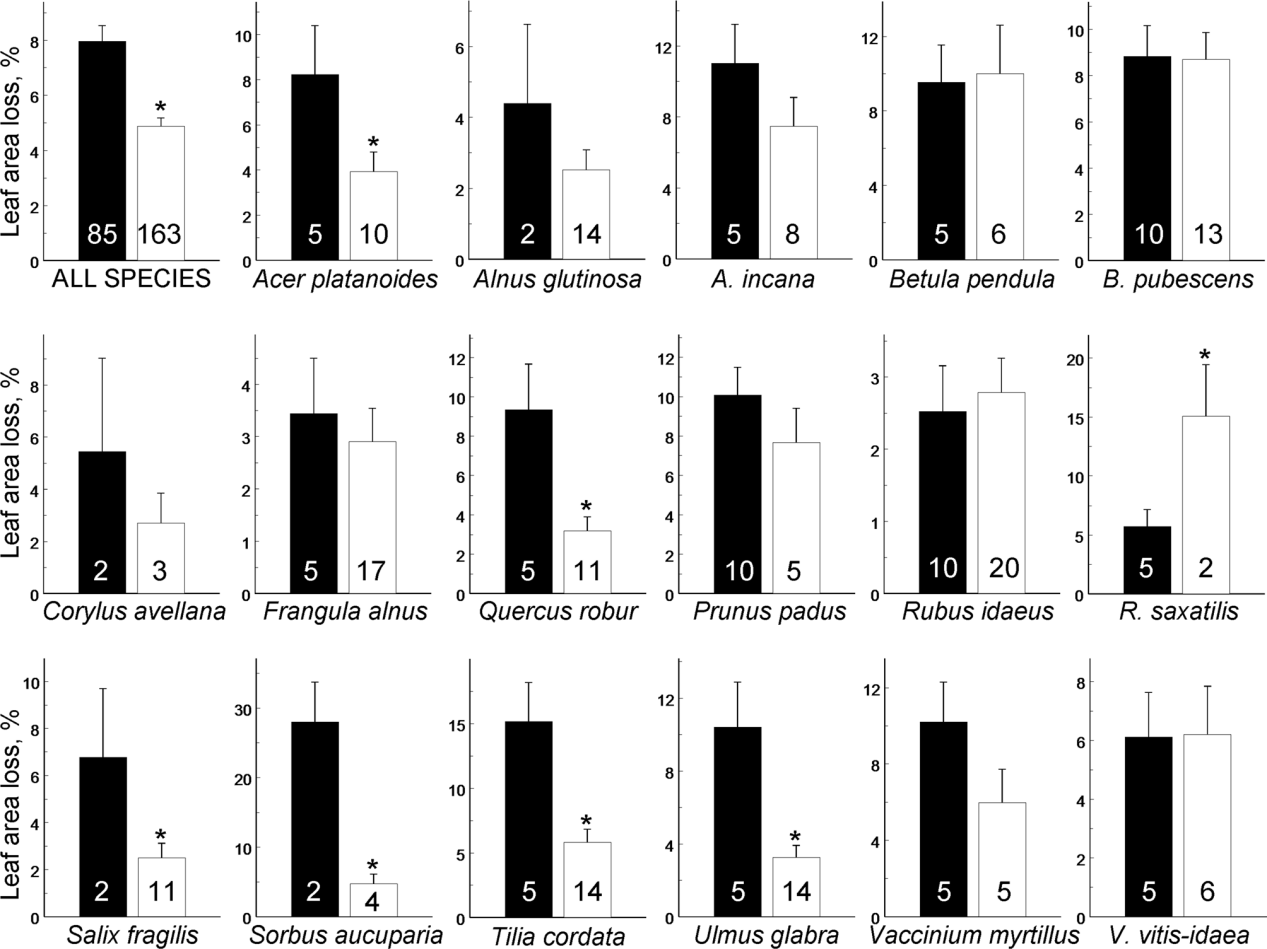
Leaf area loss (estimated marginal means + SE) measured from ecological samples (black bars) and from herbarium specimens (white bars) collected at the same time from the same localities (sample sizes are shown within bars). An asterisk indicates a significant (*P* < 0.05) difference between herbivory levels measured from these samples. Note the variation in the vertical scale.

**Figure 2:**
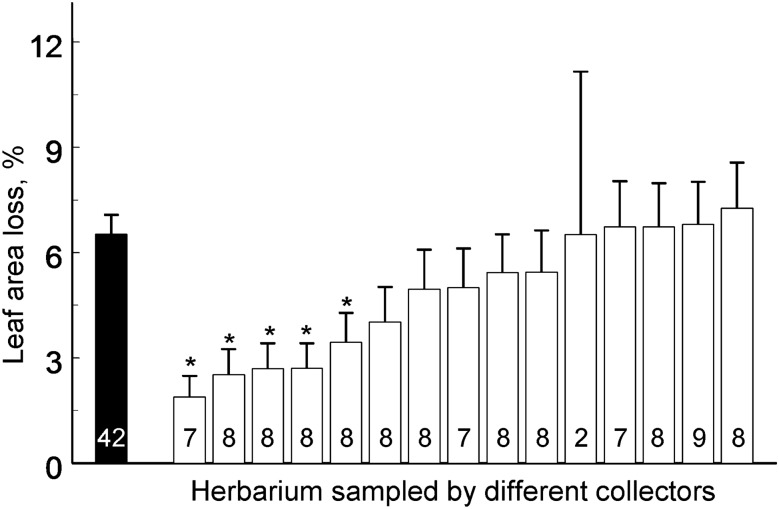
Leaf area loss (estimated marginal means + SE) measured from ecological samples (black bar) and from herbarium specimens (white bars) sampled by 15 individual collectors (sample sizes are shown within bars). An asterisk indicates a significant (*P* < 0.05) difference between the herbivory levels measured from herbarium specimens sampled by individual collectors and from ecological samples.

**Figure 3:**
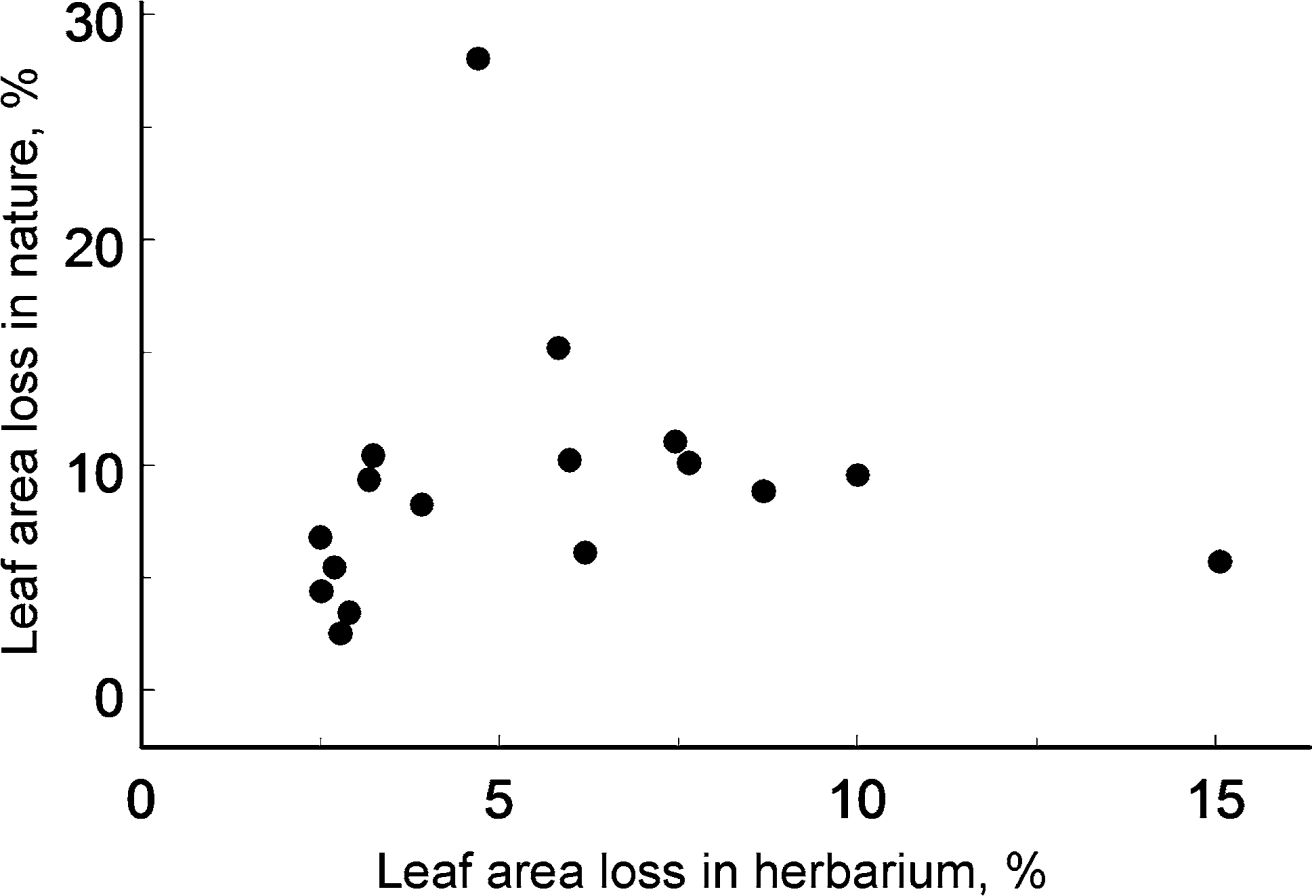
Leaf area loss in nature plotted against the average level of herbivory measured from herbarium specimens. Each circle corresponds to one of the plant species shown in Fig. 1.

The average losses of woody plant foliage (Supplementary Data S1) were significantly lower for herbarium specimens (4.87%) than for ecological samples (7.96%), although the differences between these two types of samples varied with the plant species (Table 1, Fig. 1). Collectors generally prefer branches with low levels of herbivory, and when insect damage increases in nature, the difference between herbarium specimens and ecological samples becomes greater (see e.g. *Sorbus aucuparia* and *Tilia cordata* on Fig. 1). Individual collectors significantly differed in their attitudes to leaf damage by insects (*F*_14, 79_ = 1.93, *P* = 0.04; Fig. 2) while collecting herbarium specimens, with their choices ranging from careful selection of branches with nearly undamaged leaves to taking almost no account of the extent of insect herbivory. As a result, the actual levels of herbivory and the damage in herbarium specimens varied independently from each other (regression analysis: *F*_1, 15_ = 0.07, *P* = 0.80; Fig. 3). Additional analysis showed that exclusion of *Sorbus aucuparia*, the species with the extreme differences between the levels of herbivory measured from two types of samples, did not change our main result: the correlation between the levels of herbivory in ecological samples and in herbarium specimens remained not significant (data not shown).

**Table 1:** Sources of variation in losses of woody plant foliage to insects: results of field experiment (SAS GLIMMIX procedure).

Effect type	Source of variation	Test statistics	d.f	Test value	*P*
Fixed	Site	*F*	1, 151.3	1.64	0.20
	Sampling protocol^1^	*F*	1, 12.1	10.45	0.0071
	Site × Sampling protocol	*F*	1, 45.0	2.00	0.16
Random	Plant species	*χ*^2^	1	5.28	0.0108
	Sampling protocol × Plant species	*χ*^2^	1	4.43	0.0176

^1^Herbarium vs. ecological sampling.

### Preference of press-dried plant specimens by herbarium curators

The 17 herbarium curators reported the use of the following criteria for selecting herbarium specimens for accession: good representation of morphological characters (13 respondents); high quality of preservation and preparation, including less overlapping plant parts (9 respondents); large size, in terms of the amount of material available for a study (7 respondents); low damage by insects or fungi (7 respondents); presence of additional identifiable organisms, e.g. lichens and leafminers (5 respondents); high aesthetic quality/beauty (3 respondents); and high variation in leaf size (1 respondent). Interestingly, two respondents reported a preference for specimens with leaf mines (i.e. with damage by identifiable insects) but avoidance of specimens with high damage by chewing insects (i.e. damage that cannot be associated with a particular insect species).

**Figure 4:**
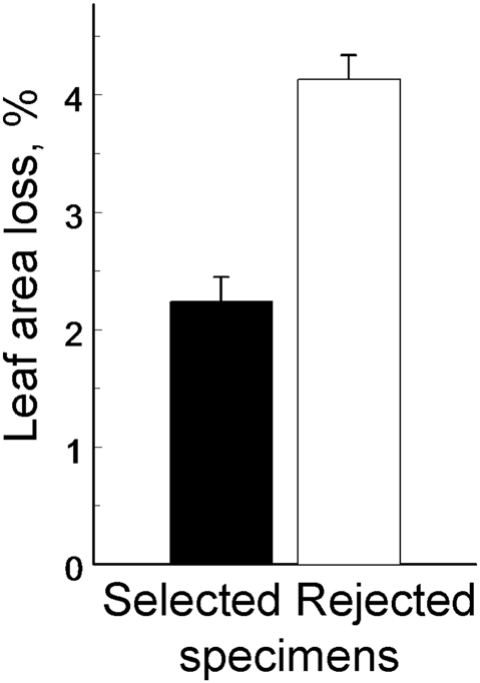
Leaf area loss (means + SE) in plant specimens that were selected (black bar) and rejected (white bar) by the persons responsible for accession/de-accession of plant specimens in various herbaria. The difference between bars is significant (*P* < 0.0001).

Two respondents refused to select between paired images, because (as they argued) all or almost all plant individuals used as test objects do not deserve accession into herbarium collections. The remaining 15 respondents (Supplementary Data S2) clearly preferred less damaged specimens (which were selected 211 times from 315 offers; the difference from the random selection: *χ*^2^ = 18.71, d.f. = 1, *P* < 0.0001). Consequently, the selected plant specimens had only 60% of the average foliar damage found in the rejected plant specimens (*F*_1, 28_ = 41.54, *P* < 0.0001; Fig. 4). The selection was independent of the position (left or right) of the more damaged plant specimen within a pair (left or right; *F*_1, 14.9_ = 0.29, *P* = 0.60).

The respondents significantly differed in their preferences towards less damaged plant specimens (*χ*^2^ = 10.15, d.f. = 1, *P* = 0.0007). The percentage of less damaged specimens selected by individual respondents from 21 pairs of images varied from 38.1 to 90.5%.

The probability of selection of less damaged specimen also varied among pairs of plant specimens (*χ*^2^ = 41.63, d.f. = 1, *P* < 0.0001). The percentage of less damaged specimens selected by 15 respondents from a pair of images varied from 0 to 100%, and in 10 pairs of images this preference was statistically significant (*P* < 0.05): in eight pairs, the respondents consistently selected the less damaged specimen, but in two pairs, they clearly preferred the more damaged specimen. The differences in the absolute levels of herbivory between the paired specimens did not explain the detected preferences (*F*_1, 13.6_ = 0.09, *P* = 0.77).

### Discussion

Collection of multiple specimens of the same species in the same locality is often performed when inventorying vegetation of a certain region. Collection of duplicates is also recommended by some manuals^30^. Therefore selection among several specimens collected in the course of field excursions is a part of herbarium practice^31^, and our experiments mimic this practice in as many details as possible.

The results of our two experiments consistently demonstrated that measurements of losses of woody plant foliage from herbarium specimens strongly underestimate the levels of background insect herbivory observed in nature, thus confirming our first hypothesis. This finding is not surprising, because many textbooks, both old and recent^13,15–17^, advise the collection of undamaged plants and plant parts for herbaria. The likely underestimation of actual herbivory has been mentioned in previous studies exploring herbarium specimens^9,10,24^, but we now have provided the first quantitative estimate: the combined preferences of the specimen collectors and the herbarium curators decrease the level of background foliar damage of herbarium specimens, on average, to about a half the level observed in nature at the time of sampling.

The overall underestimation of herbivory due to the non-random sampling of herbarium specimens would not create a major problem if the differences in herbivory between samples obtained from the same site by two different methods (botanical and ecological) remained constant. However, this is not the case, because, as shown in our study, the increase in levels of leaf damage by insects in nature was not followed by proportional increase in herbivory measured from herbarium specimens. In other words, collectors dampen the differences in herbivory between more and less damaged plants; consequently, analysis of herbarium specimens does not allow a distinction between situations when plant damage in nature is low and of no concern to the collector (see, for example, *Rubus idaeus* in Fig. 1) and when the damage in nature is rather high and forces the collector to search for specimens showing the least damage (see, for example, *S. aucuparia* in Fig. 1).

Moreover, one plant species collected by us (*Rubus saxatilis*) showed the opposite pattern: the leaves of herbarium specimens demonstrated 2.5-fold higher losses to insects when compared with the ecological samples (Fig. 1). We explain this exceptional pattern by different handling of the runners (the long creeping shoots) of this species. The collectors chose only vertical (fruiting) stems with 3–4 leaves and discarded the long (up to 2 m length) runners, presumably because they did not fit the size of the herbarium sheets. By contrast, the ecological samples included these long runners. The leaves of runners are younger than the leaves of vertical stems, and these young leaves suffered less damage due to their shorter exposure to herbivores. Consequently, exclusion of runners from the herbarium specimens caused an overestimation of the plant-specific leaf damage. This example suggests that collector’s preference may be influenced by plant species traits.

Thus, not only the magnitude but even the direction of the effects of non-random sampling of herbarium specimens on measures of herbivory may change with the plant species (Fig. 1). Consistently, we found that regression analysis, contrary to our second hypothesis, cannot be used to estimate field herbivory from the herbarium specimens, even before the specimens underwent additional selection by the curators for inclusion in the herbaria. The inclusion step then further affects the level of herbivory observed in herbarium specimens by deciding their fate during accession and de-accession procedures.

Our conclusion regarding impossibility to deduce the levels of insect herbivory occurring in nature from herbarium specimens is based on the analysis of the variation in herbivory among plant species, which were collected at the same time. However, a space-for-time substitution is commonly used as an alternative to long-term studies in global change research^32^. Consequently, our results suggest that in the years with high herbivory the botanists are more selective against damaged plant species than in the years with low herbivory. The effects of this presumed among-year variation in the strength of selection for less damaged plant specimens on long-term herbivory data extracted from herbaria remain to be studied.

An earlier study^10^ reported non-significant variation in herbivory between the samples of different collectors. However, the cited study involved plants that had been sampled at different sites and on different dates, and this spatio-temporal variation was likely to prevent detection of the preferences of individual collectors with respect to plant damage. Furthermore, the subsequent accession/de-accession preferences of curators were also not taken into account. By contrast, in both our experiments, the collectors simultaneously sampled the same plant species at the same site and the curators evaluated the same set of plant images. This study design revealed a significant variation in attitudes of both collectors and curators towards plant damage by insects.

Our first experiment involved botany students who were qualified for sampling of plants for herbarium collections, whereas the second experiment involved experienced botanists. In our opinion, it is unlikely that more experienced collectors are less selective in respect of collected plant specimens than the botany students. The differences in herbivory between herbaria and ecological samples would rather increase with the increase in collector’s experience.

The strength of the curator’s impact on the fate of damaged plant specimens would depend on the policies of a particular herbarium and on the individual preferences of the curator, as well as on the type of plant damage by insects. In particular, the presence of leaf miners, even in high numbers, facilitated the preference of a plant specimen by some curators, whereas other curators considered leaf mines to be ordinary damage that diminished the value of the plant specimen. Similarly, branch infestation by scale insects documented from herbarium specimens^33^ may also overestimate the infestation occurring in nature, because some curators reported a positive attitude towards additional, identifiable organisms associated with herbarium specimens. A disproportionately large percentage of specimens deposited in individual herbaria have been collected by a very few individuals, termed mega-collectors^19^, and most herbaria have only a few curators. Therefore, the combined impacts of mega-collectors and curators, with their associated preferences and idiosyncrasies, may end up shaping the patterns of collection bias in each individual herbarium in a truly unpredictable manner. This variation can be neglected only when analysing very large samples taken at random from a number of different herbaria.

To conclude, cross-validation with field data demonstrates the need for correcting for underestimates (or overestimates) of herbivory that arise from the use of herbarium collections due to variations associated with the levels of herbivory, the plant species traits, and the directions and strengths of preferences for less or more damaged plant specimens by both the collectors and curators. In terms of overall losses of leaf area to insects, herbaria may act as distorting mirrors and further studies are needed before they could serve as reliable sources of information about historical levels of insect herbivory.

## Methods

### Effect of sampling protocol on leaf area lost to insects

Herbarium specimens and ecological samples aimed at evaluation of insect herbivory were collected in two localities: a forest in Lembolovo near St Petersburg (60°23′33"N, 30°15′54"E) and a park at the St Petersburg State Forest Technical University (59°59′28"N, 30°20′36"E). At both sites, plants were collected from an area of approximately 0.1 km^2^. The sampling was performed during the second half of August 2018, when most plant-feeding insects had completed their development; therefore, the measured leaf damage had accumulated during the entire growth season.

The study was carried out in accordance with “The ethical principles of research with human participants and ethical review in the human sciences in Finland (the Finnish National Board on Research Integrity TENK guidelines 2019)”. According to this document, our research did not require ethical review. The participation of students and herbaria curators in our study was voluntary, and the participants were informed that plant samples collected in the course of this study and the results of plant quality evaluation will be anonymously used in scientific research.

Herbarium specimens were collected by two groups of students (supervised by A.A.E. and M.Y.G.) as a part of their field courses in botany. Neither supervisors nor students were informed about the goals of the study. Each student was instructed to collect one branch of each woody plant species native to the study region and to press-dry it for future deposition in the herbarium; thus, the size of the collected branches was limited by the size of paper used to mount herbarium specimens (297 × 410 mm). The information provided to the supervisors and students prior sampling of herbarium specimens is presented in Supplementary Methods S1.

The lists of the plant species collected by students (all plant names are given according to The Plant List^34^) were provided to M.V.K. who, jointly with V.Z., performed sampling of plant foliage in the specified localities following the protocol used in earlier studies of insect herbivory. This protocol (Supplementary Methods S2) was developed to minimise the probability of obtaining a biased estimate of foliar damage. Plant individuals were chosen haphazardly, on a ‘first found, first sampled’ basis; plant branches (or patches of dwarf shrubs) were selected from a distance of 5–10 m (i.e. from a distance that did not allow evaluation of foliar damage prior to sampling). Although both collectors were aware of the research hypothesis, the recent meta-analysis^12^ demonstrated that this knowledge was unlikely to cause bias at the stage of tree or branch selection. The levels of damage of herbarium specimens were unknown to M.V.K. and V.Z. at the time of collection of the ecological samples.

The average sample sizes used by us (five and ten individuals per plant species for ecological and herbarium samples, respectively) are typical for a wealth of published studies: the median value of sample size, calculated across 309 publications on insect herbivory, was five individuals per plant species^35^.

The percentage of leaf area lost to insects was measured using all leaves from the sampled branches (including the petioles of completely consumed leaves). Following a widely used methodology^36,37^, each leaf was assigned to one of the damage classes according to the percentage of the area of the leaf lamina that had been consumed or damaged by insects: intact leaves, 0.01–1, 1–5, 5–25, 25–50, 50–75 and 75–100%. This assessment combined the damage imposed by defoliators, miners and gallers; it was conducted by M.V.K. The visual assessment of herbivory is less laborious than the analysis of leaf images, which, in particular, requires manual reconstruction of the edge of each damaged leaf. However, both methods give similar results in terms of the accuracy of measurements^38^ and the size of the reported effects of different environmental factors on insect herbivory^12^. The possibility that the measured values were affected by confirmation bias was avoided by having two randomly selected subsamples (20 plants in each) additionally assessed by a person (J. Rikus) who was not aware of the sample origin or the research hypothesis.

To obtain sample-specific percentage of leaf area lost to (or damaged by) insects, the numbers of leaves in each damage class were multiplied by the respective median value of the damaged leaf area (i.e. 0 for intact leaves, 0.5% for the damage class 0.01–1%, 3% for the damage class 1–5%, etc.); the obtained values were summed for all damage classes and divided by the total number of leaves (including undamaged ones) in a sample^37^.

### Preference of press-dried plant specimens by herbarium curators

For this test, we used 21 pairs of woody plant specimens, each pair belonging to a different species (all collected by M.V.K. from different localities: Turku, Finland; Lava River, Russia; Inverness, Scotland; Batumi, Georgia). The specimens within each pair were selected to represent different types and different levels of leaf damage by insects. The damaged leaf area in these specimens was measured as described above; it varied from zero to 16.9% (mean ± SE: 3.20 ± 0.69%, n = 42 specimens), i.e. it was within the range of values typical for background insect herbivory^35^. The difference in insect damage between the paired specimens ranged from 0.2 to 15.7%. The plants were scanned, and their colour images were offered to respondents as PowerPoint presentations (Supplementary Methods S4). The position (left or right) of the more damaged specimen within a pair was randomised.

Our aim was to obtain the opinion of respondents who were either the official herbarium curators or other scientists who work with plant collections (for simplicity, all these respondents are collectively referred to as “curators” elsewhere in the text). The respondents were drawn from each of the 32 largest herbaria in the world with collections amounting to at least 2 million specimens^39^. The names of the respondents were haphazardly selected from the Index Herbariorum^40^ and/or from web-pages of individual herbaria. All respondents were informed that we were exploring factors that affect the selection of certain plant specimens for long-term storage in herbaria. To avoid prejudice, we did not mention that our focus was on insect damage. We asked each respondent (1) to select one specimen from each pair for placement in the main collection of their herbarium; and (2) to list the criteria that they had used to select specimens for accession into the permanent collection. We sent 49 requests and received 17 responses (Supplementary methods S3).

### Data analysis

We modelled the proportion of insect damage in leaves using a generalized linear mixed model approach (SAS GLIMMIX procedure^41^) with a beta error distribution (which is a continuous probability distribution, so it fit here to the percentage data for the interval 0–100%) and a logit link function^42^. In this model, the study site (Lembolovo vs St Petersburg), sampling method (herbarium vs ecological sampling) and their interaction were considered fixed effects, while plant species (17 in total) and their interaction with the sampling method were treated as random effects. We facilitated accurate *F *tests of the fixed effects by adjusting the standard errors and denominator degrees of freedom^43^. The significance of random effects was explored by a likelihood ratio test^44^. The same model, with the addition of one more fixed effect (collector), was used to test for the variation among students in their preference/avoidance for less damaged plant specimens. The results of two herbivory measurements conducted by different persons were compared with a paired *t* test. The possibility to deduce the actual levels of herbivory from data obtained by studying herbarium specimens was explored by regression analysis of the species-specific values of leaf losses to insects (SAS REG procedure^41^).

The preferences/avoidances of our respondents with respect to plant damage by insects, based on their selection of one image from each pair (binary distribution: ‘more damaged’ vs ‘less damaged’), were logit-transformed and analysed by SAS GLIMMIX procedure. We checked whether this selection was influenced by the position of a more damaged plant within a pair (left or right; fixed effect) and asked whether the respondents (considered as random effects) showed different preferences/avoidances and whether these preferences/avoidances depended on the absolute difference in leaf damage between the two compared specimens (considered as covariate). We also used frequency analysis to explore the deviation from random (1:1) choice and one-way ANOVA to compare the leaf losses to insects between the selected and rejected plant specimens.

## Supplementary information

Supplementary file1

## Data Availability

All data from this study are included in this publication and its Supplementary Material.
