# Supplementary material for: Biases in estimation of insect herbivory from herbarium specimens
Source: Sci Rep. 2020 Jul 23;10:12298. doi: 10.1038/s41598-020-69195-5 (PMC7378164; doi:10.1038/s41598-020-69195-5)
Supplement: Supplementary file 1 — Supplementary file1 [file 41598_2020_69195_MOESM1_ESM.pdf]

Biases in estimation of insect herbivory from herbarium specimens

Mikhail V. Kozlov[1*], Irina V. Sokolova[2], Vitali Zverev[1], Alexander A. Egorov[3], Mikhail Y. Goncharov[4], and Elena L. Zvereva[1]

[1] Department of Biology, University of Turku, 20014 Turku, Finland

[2] Herbarium, V. L. Komarov Botanical Institute, Professora Popova Str. 2, 197376 St. Petersburg, Russia

[3] Department of Biogeography and Nature Preservation, Institute of Earth Sciences, St. Petersburg State University, Universitetskaya nab. 7–9, 199034 St. Petersburg, Russia

[4] St. Petersburg Chemical-Pharmaceutical University, Professora Popova Str. 14, 197022 St. Petersburg, Russia

*Correspondence author. E-mail: mikoz@utu.fi. ORCID: 0000-0002-9500-4244.

Supplementary information

Supplementary Methods S1

Information provided to supervisors of students who collected herbarium specimens (translated from Russian)

The students are assumed to be familiar with the basic rules of collecting plants and mounting herbarium specimens and to know that the size of the paper used for herbarium specimens is A3. Nevertheless, before initiating the sampling, the students should be reminded of the importance and value of herbarium specimens, as well as about the long lives of herbaria (consider mentioning the herbarium of Linnaeus), which are used by many generations of scientists. The students should believe that the samples they collect will become herbarium specimens destined for a long life.

Each student should be requested to collect a single specimen of each native (i.e. not introduced) species of leaf-bearing woody species within a clearly defined area (approximately 4−10 ha) of a forest or park. The teacher should use natural, easily distinguishable borders (railways, roads, forest edges, lake shores, etc.) to outline the sampling area. The students should be informed that collection of plants outside the specified

area is unacceptable[1].

Inform the students about the number of species of leaf-bearing woody plants that can be found in the study area, and remind them that dwarf shrubs (bilberry, blueberry etc.) are also classified as woody plants. Also indicate the most common species that do not belong to the local flora and therefore should not be collected.

Students should be ordered to work individually; that is, each student should collect plants on their own (not as a group). A desirable number of specimens is 12–15 of each of 7–8 woody plant species (totalling approximately 100 specimens). Thus, if the group includes 15 students, each student should be asked to collect 7 plant specimens; if the group includes 20 students, each student should be asked to collect 5 plant specimens, and so on.

Upon completion of sampling, the teacher will go over the collected samples to check for (1) the presence of introduced species; if any specimen of a non-native plant is found, it is trashed, and the student is ordered to collect a specimen of another (native) species that is not already present in their collection; (2) the size of the collected specimens; if any specimen is too small relative to the size of herbarium sheet (A3), the student is ordered to collect a larger specimen of the same species.

The teacher should reject only non-native species; there are no other grounds for rejection of specimens collected by students. The specimens which are “not typical”, dirty, damaged, without flowers and fruits, etc. will remain in the herbarium on the same rights as “ideal” specimens. The teacher may explain to a student the shortcomings of the collected material but should not require re-sampling.

The collected specimens should be press-dried. Mounting of samples on herbarium sheets is not required (but can be done). The label of each specimen should include the name (or other identifier) of the collector. When the sampling is completed, the teacher is expected to immediately supply M. Kozlov with (1) the list of all species of woody plants collected by students; and (2) a map showing the exact boundaries of the site from which the plant specimens were collected. The samples should be transferred to M. Kozlov after the drying process is complete.

[1] This statement was necessary to ensure that ecological records were made from exactly the same area, from which herbarium specimens have been collected.

Supplementary Methods S2

Protocol used to collect samples for measurements of leaf losses to defoliating insects in ecological research

The choice of plant individuals and branches should be random. Therefore, choose an individual of the target plant species which is growing at least 5 m apart from you, and choose a branch that you will sample. It is critically important that you point at the branch from at least 5 m distance, and that you do not change your choice later on – even if you found this branch dirty or damaged when you approach the plant.

Cut the selected branch in such a way, that it bears about 50 leaves. If the selected branch has less than 50 leaves, then cut the additional branch which is next to the branch you have selected. Repeat these procedures until you collect samples from five individuals of each target species.

Collect all leaves from each branch into individual plastic bag. Do not trash any leaf, even if it is heavily injured or dirty. If some pest had completely eaten a leaf – collect leaf remnants (for example, a petiole).

Supplementary Methods S3

Staff members of different herbaria who participated in selection of plant images and/or formulated selection criteria used in accession/de-accession of herbarium specimens

Rani Asmarayani, Herbarium Bogoriense, Bogor, Indonesia;

Marie Briggs, Royal Botanic Gardens, Kew, U.K.;

Bruce G. Baldwin, Jepson Herbarium, University of California, Berkley, U.S.A.;

Hans-Joachim Esser, Botanische Staatssammlung Muenchen, Germany;

Mats Hjertson, Uppsala Universitet, Sweden;

Zdenek Kaplan, Institute of Botany, The Czech Academy of Sciences, Pruhonice, Czech Republic;

Anant Kumar, Central National Herbarium, Botanical Survey of India, Kolkata, India;

Artem Leostrin, V. L. Komarov Botanical Institute, St. Petersburg, Russia;

Caroline Loup, Université de Montpellier, France;

Sergey Mosyakin, M.G. Kholodny Institute of Botany, Kiev, Ukraine;

Chiara Nepi, Museo di Storia Naturale, Università di Firenze, Firenze, Italy;

Christine Niezgoda, Field Museum of Natural History, Chicago, U.S.A.;

Piet Stoffelen, Meise Botanic Garden, Meise, Belgium;

Henry Väre, Natural History Museum, University of Helsinki, Finland;

Ernst Vitek, Naturhistorisches Museum Wien, Austria;

Robert Vogt, Botanischer Garten und Botanisches Museum Berlin-Dahlem, Freien Universität Berlin, Germany;

Jan Wieringa, Naturalis Biodiversity Center, Leiden, Netherlands.

Selection of herbarium specimens

Joint project of the University of Turku (Finland) and V. L. Komarov Botanical Institute (St. Petersburg, Russia)

Introduction

- This presentation contains 21 pairs of images of plant specimens. The specimens within each pair belong to the same species and were collected in the same locality on the same date.
- Please assume that you are selecting plant specimens from extensive field samples for your herbarium. You are limited by both storage space and workforce; this means that you can add only one specimen from each pair to your collection.
- Please compare the images within each pair (please use a large, at least 11 inch, monitor and magnify the image to see the details if necessary) and delete the image of the specimen that you would discard. (To delete the image, return to Normal view, click on the image with the left mouse button and press “Delete” on the keyboard.) After you do that, each slide will contain only one image, showing the plant specimen that you have selected for your herbarium.
- We would find it most helpful if, on the final slide, you could list the criteria you used to select between the two specimens within each pair, and if you could answer a couple of questions about your experience in plant sampling and in curatorial work. When processing and storing the data, we will not link your responses to your name.
- Please save the edited version of the presentation and return it to Mikhail Kozlov (mikoz@utu.fi). If you do that, we will assume that you will allow us to include your name and affiliation in the list of experts who contributed to our project (to be published as electronic supplementary material to a forthcoming paper).

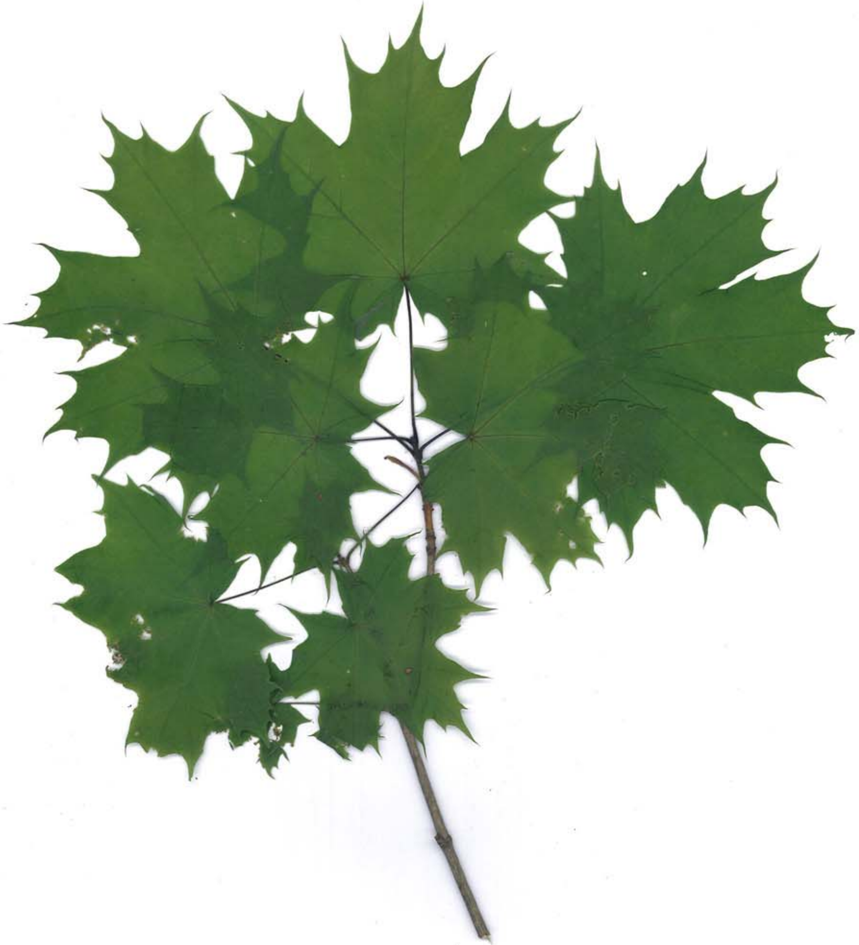

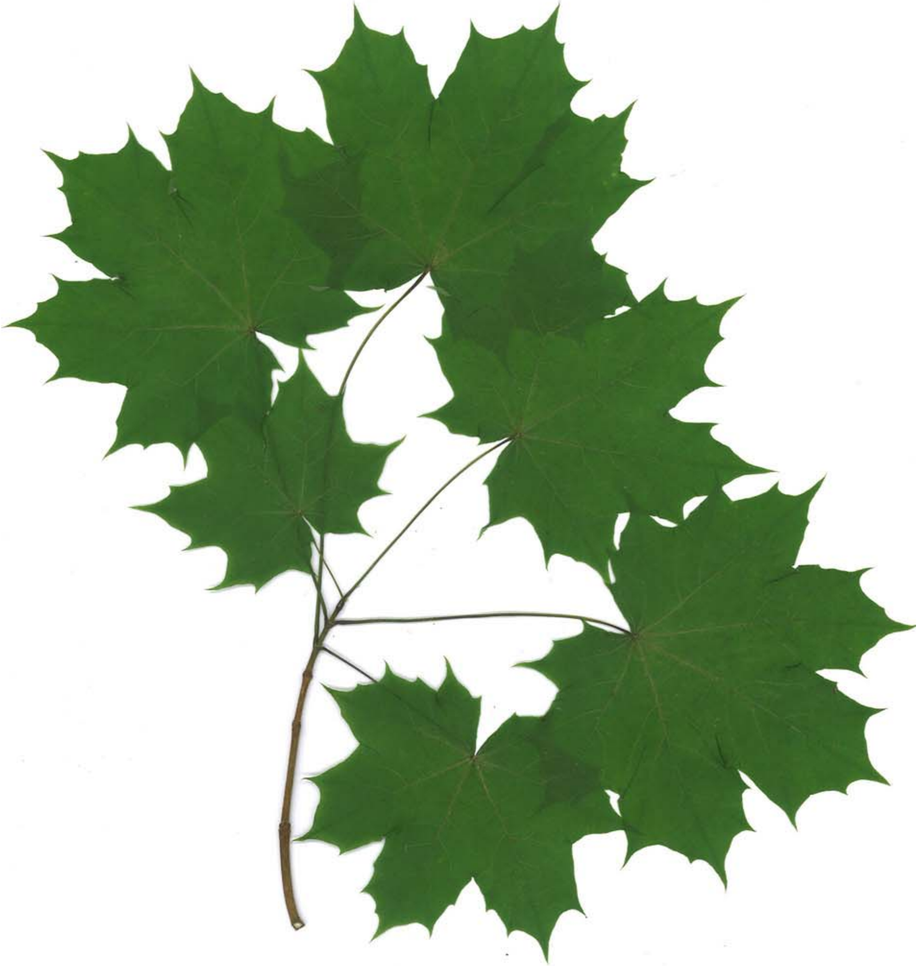

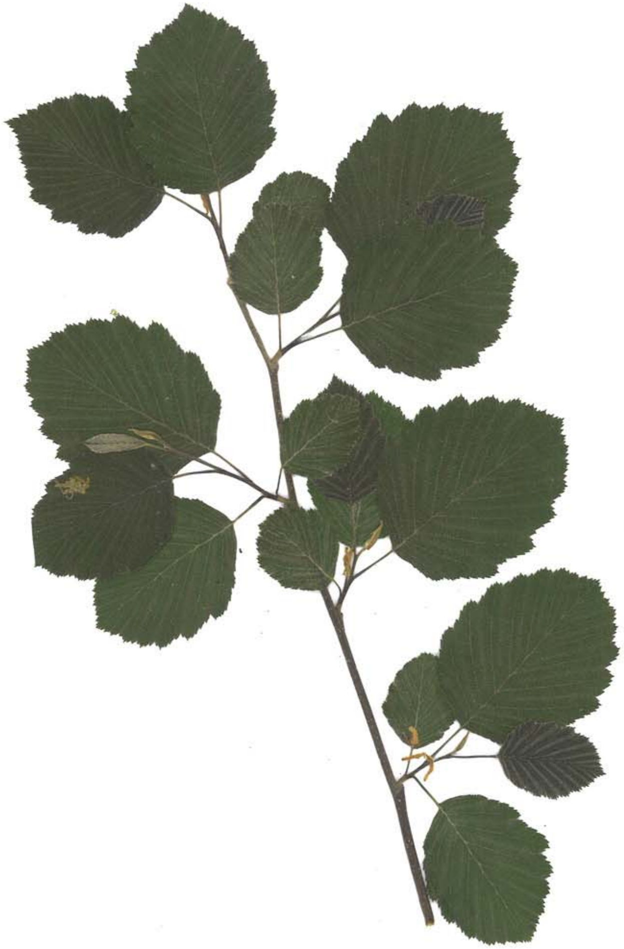
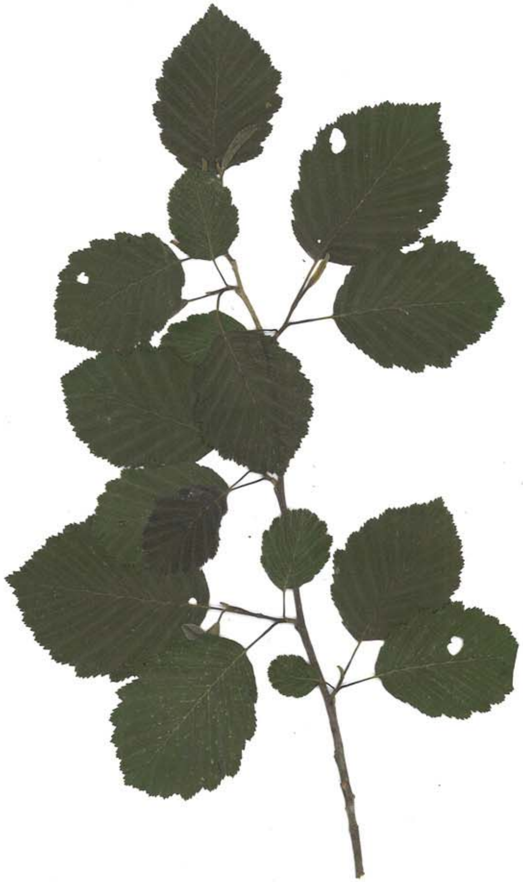

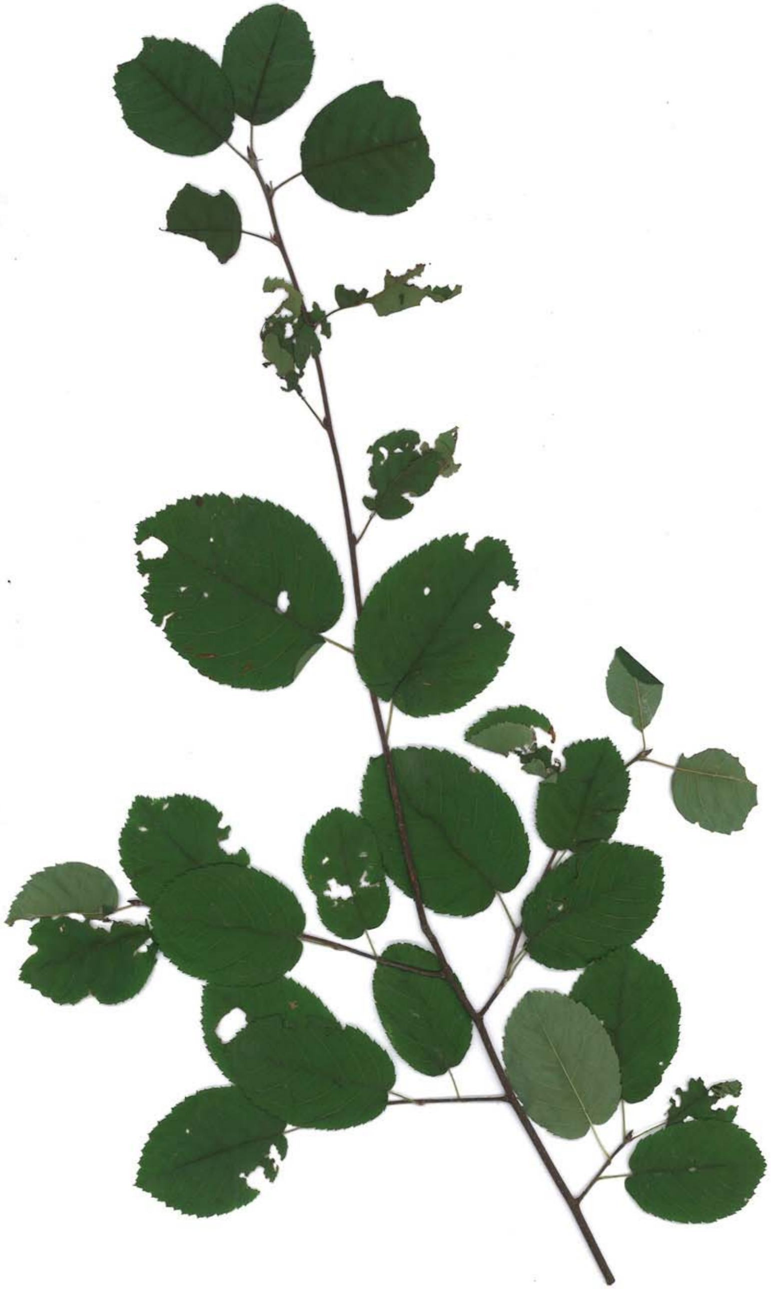
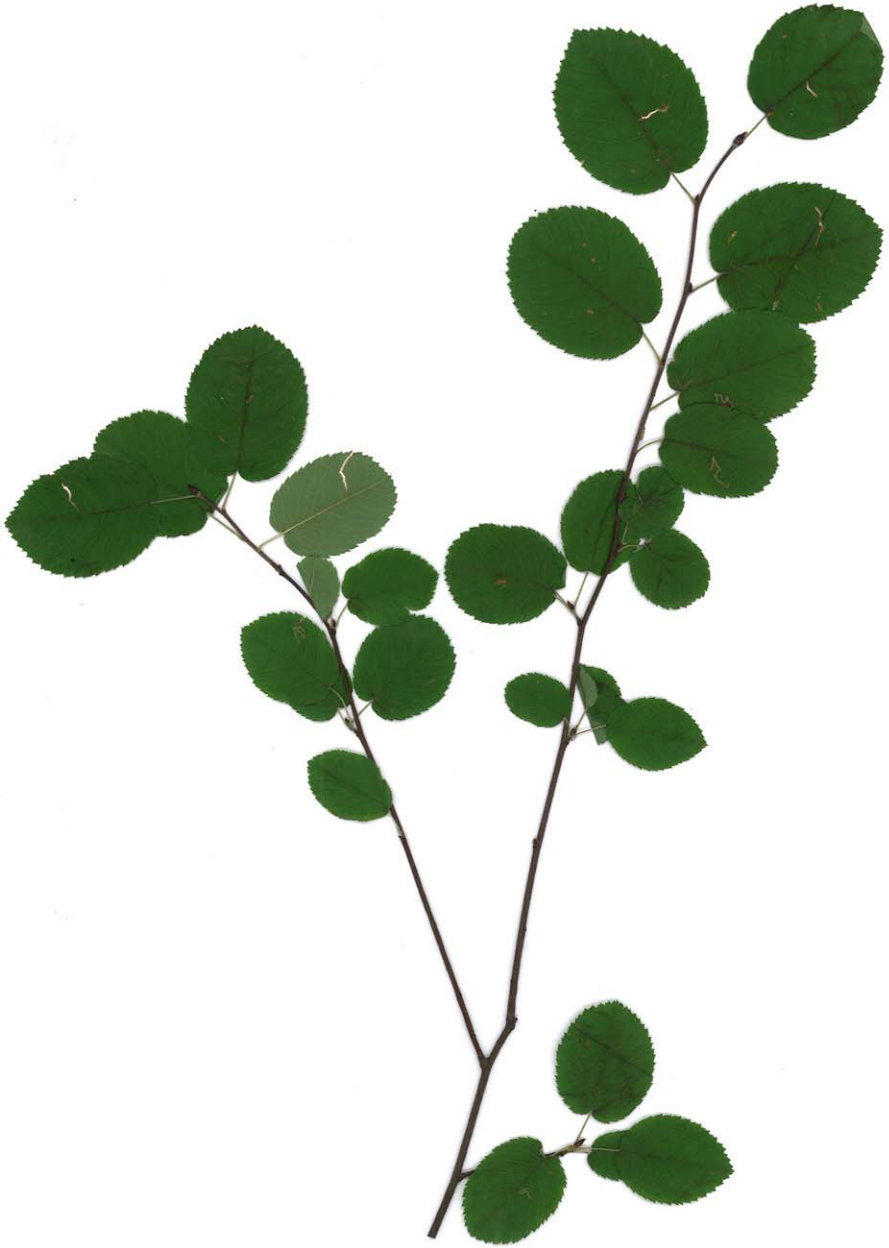

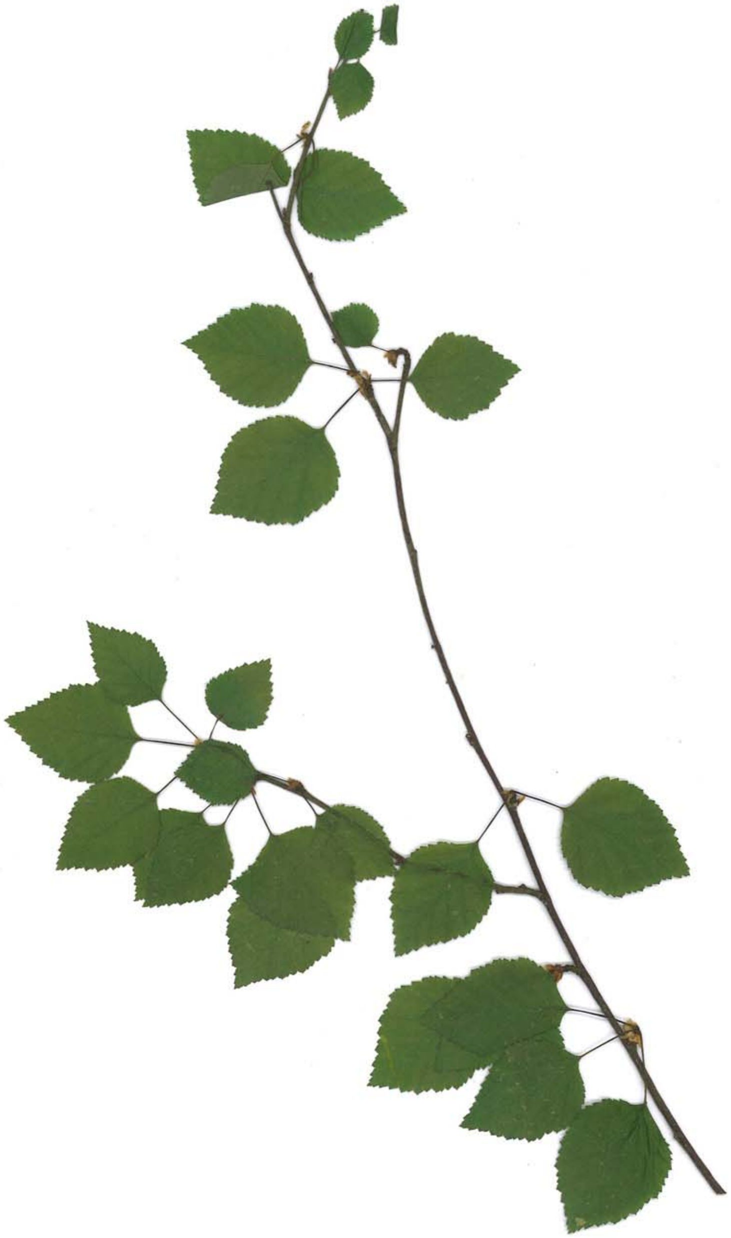
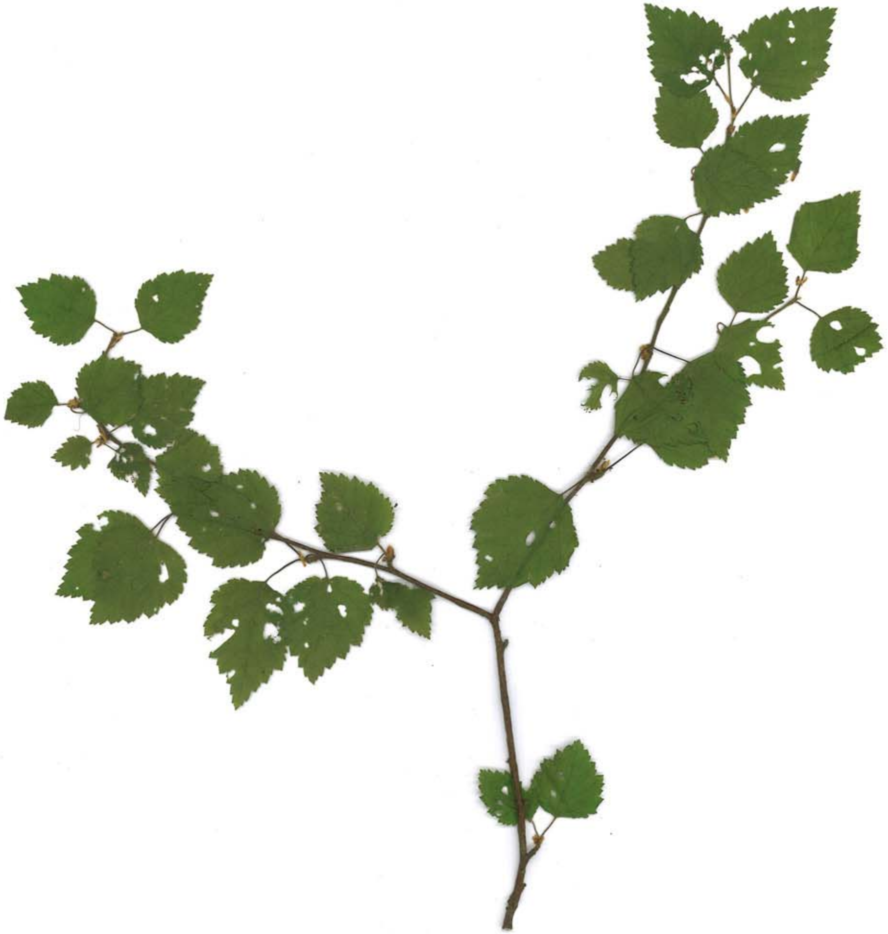

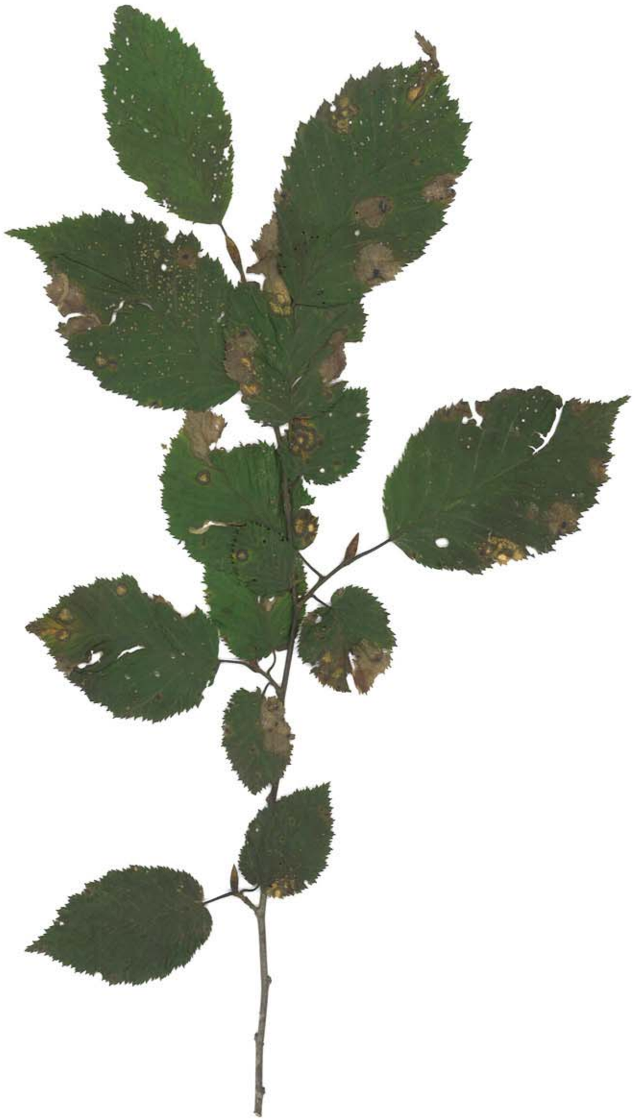

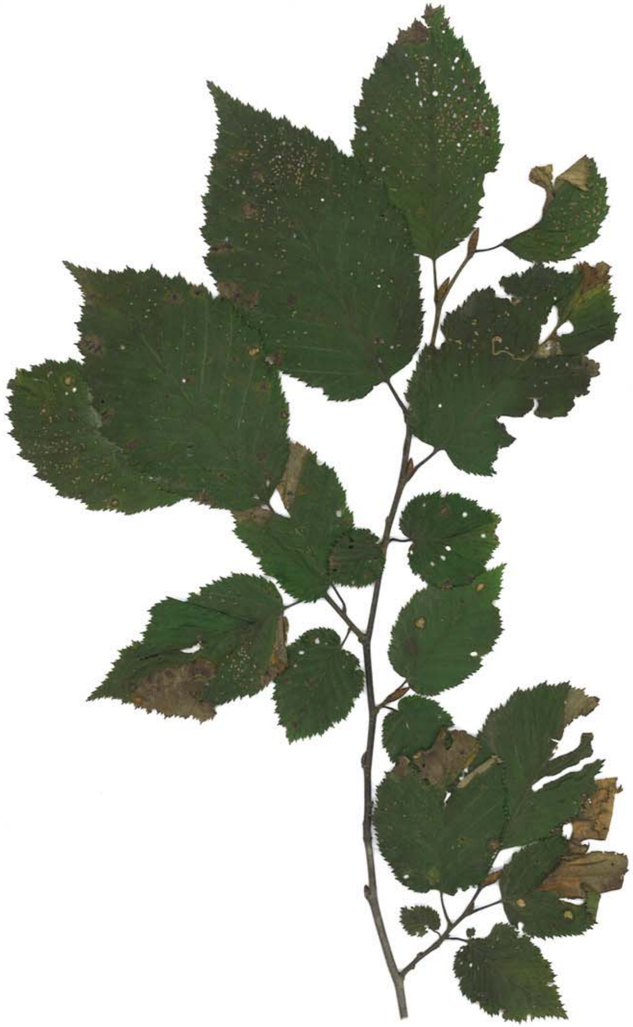

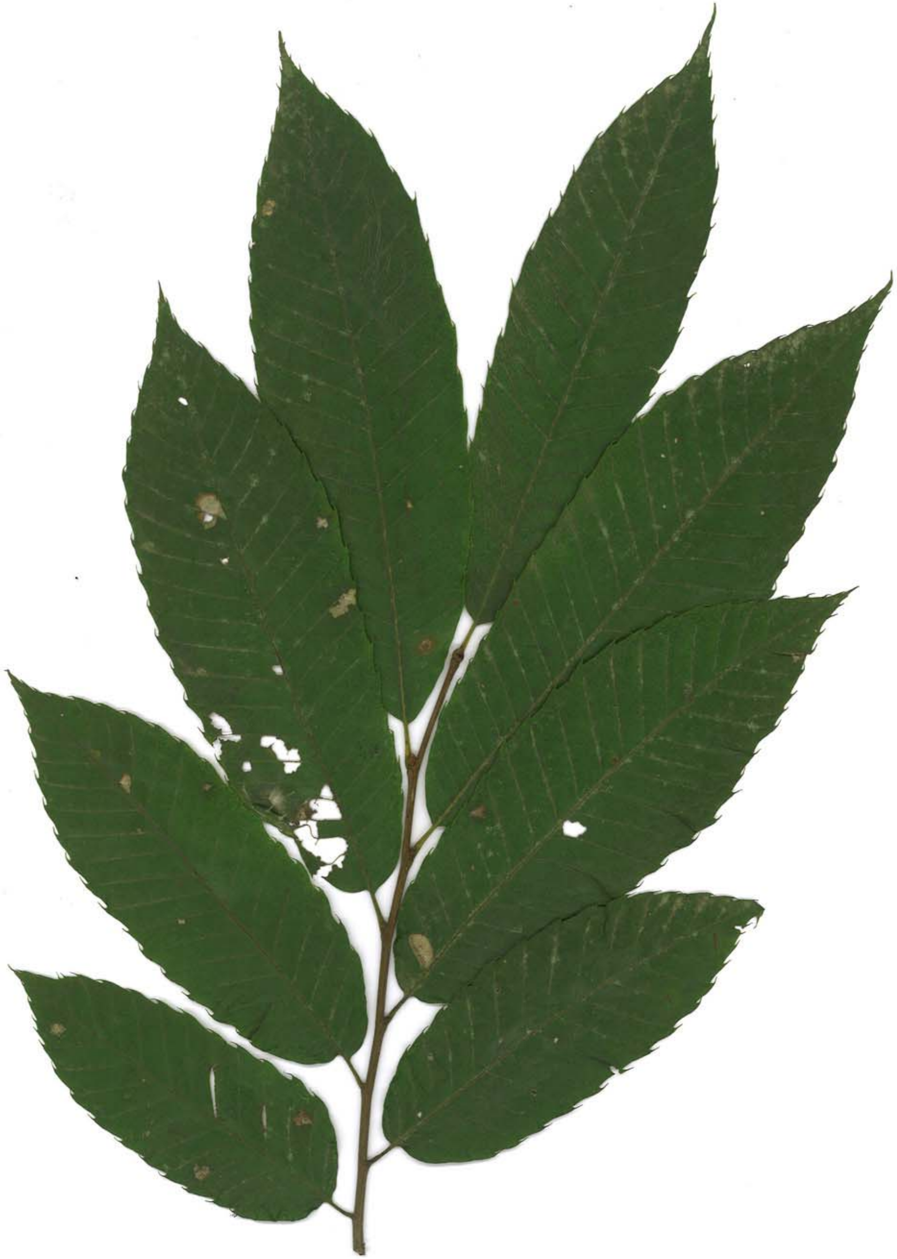
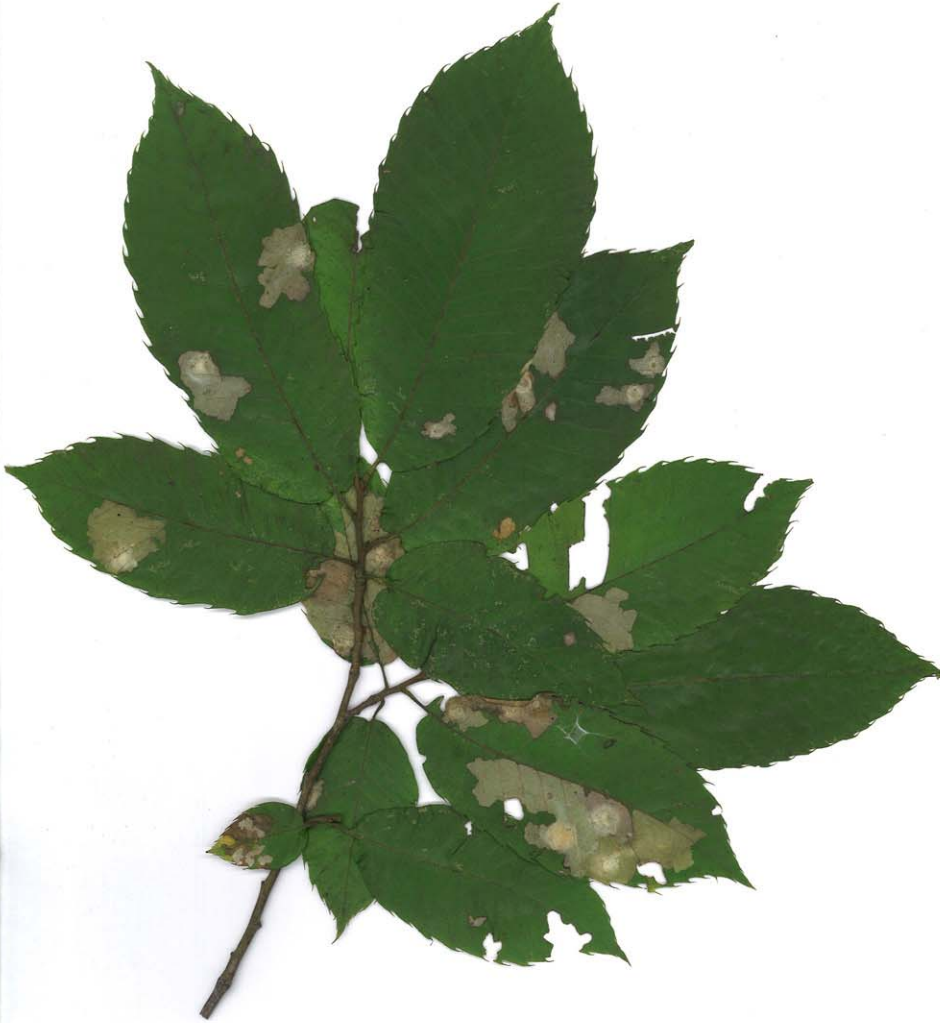

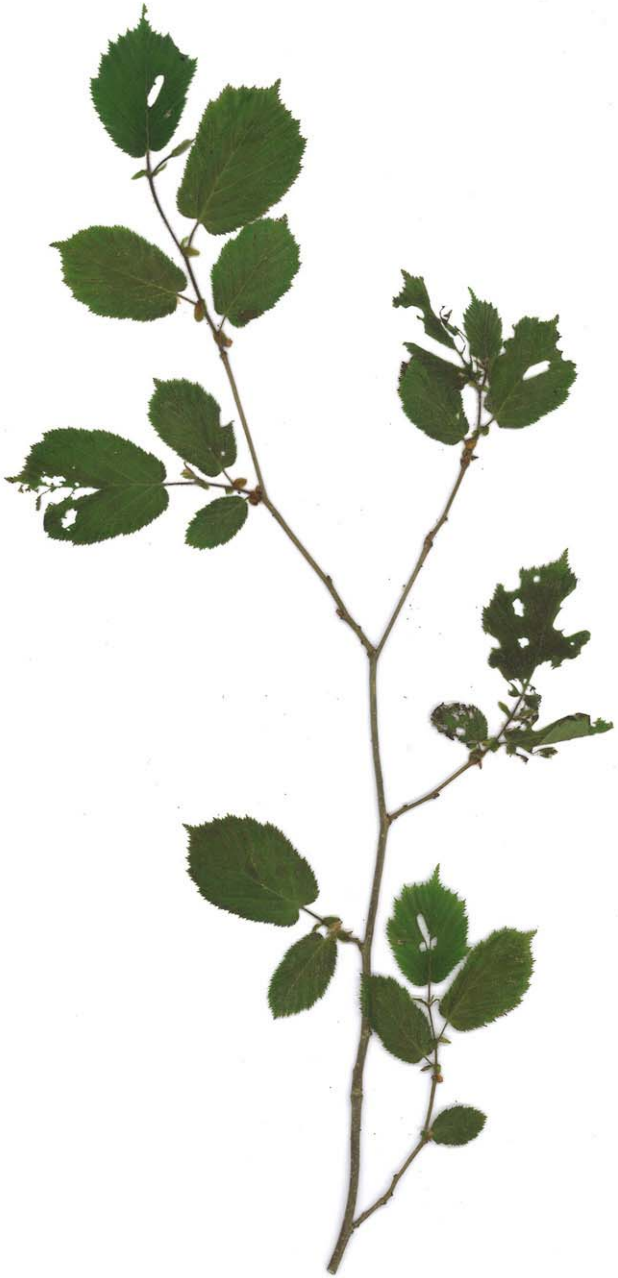

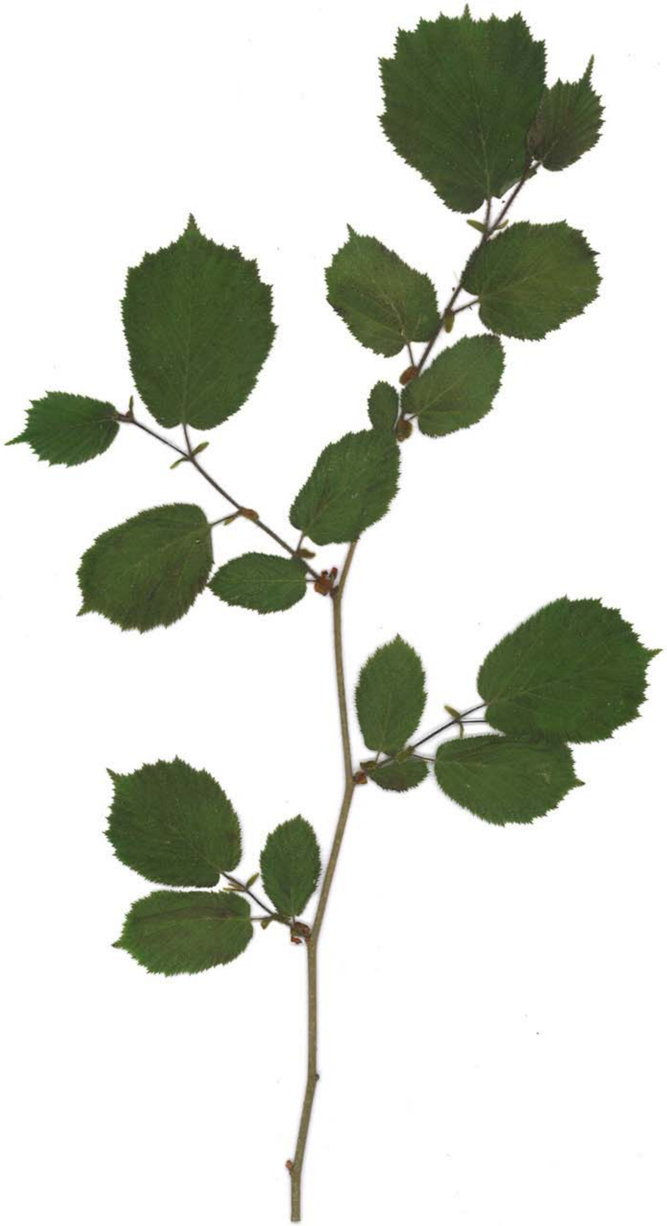

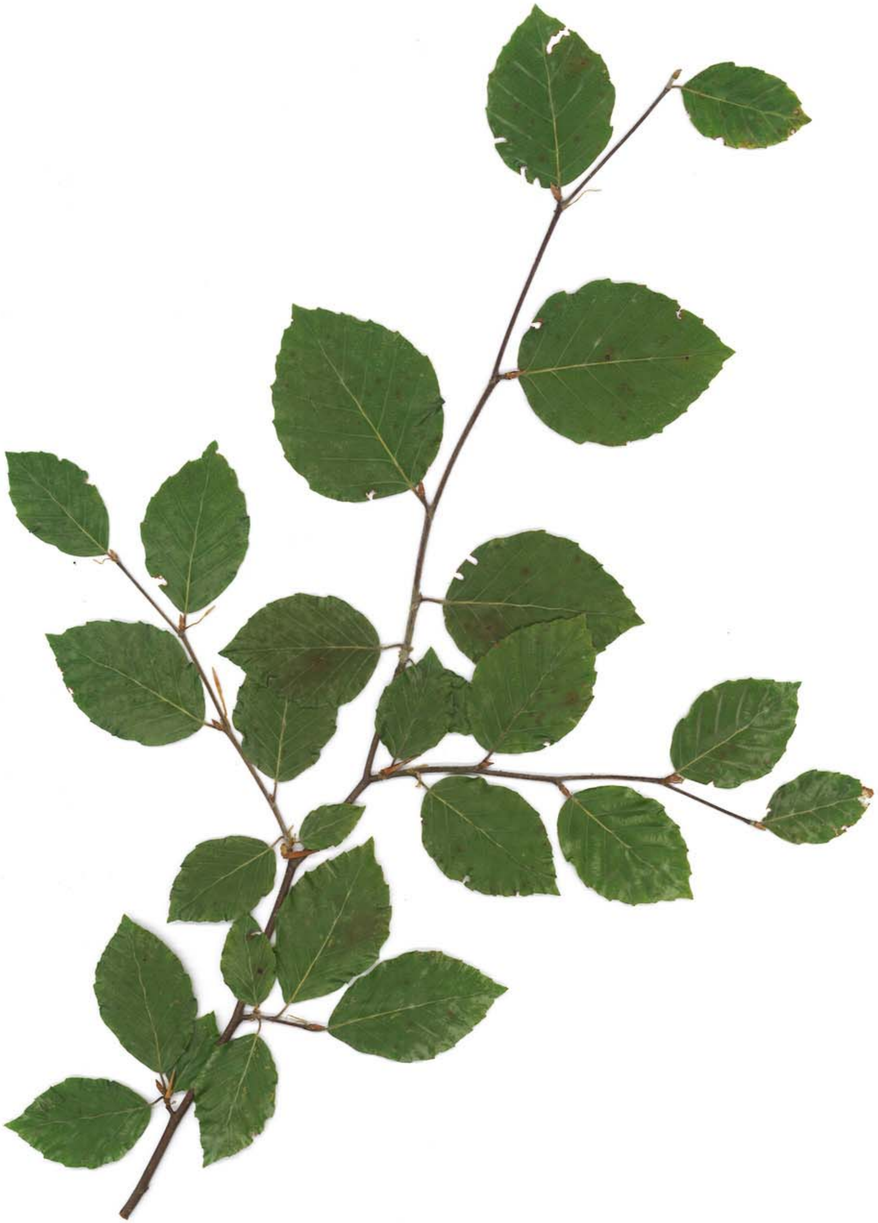
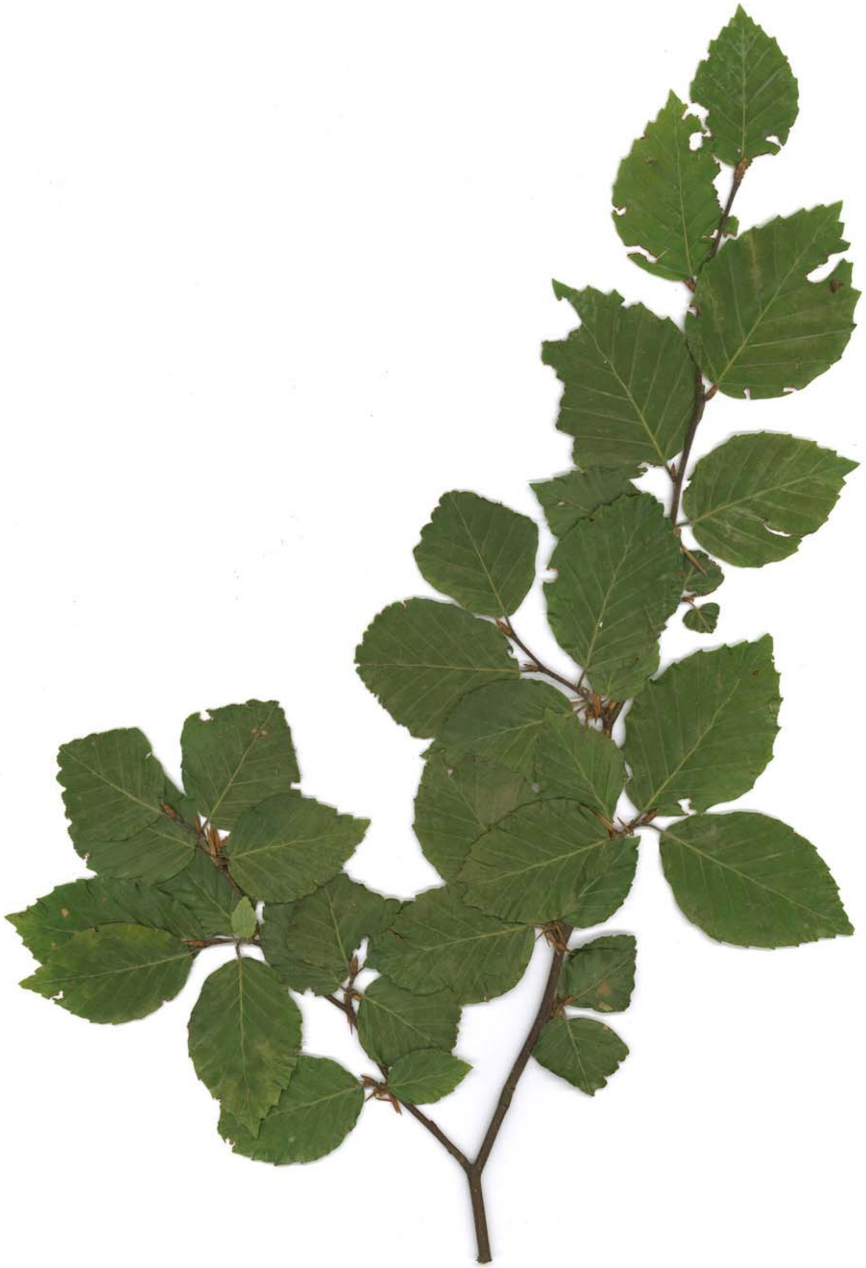

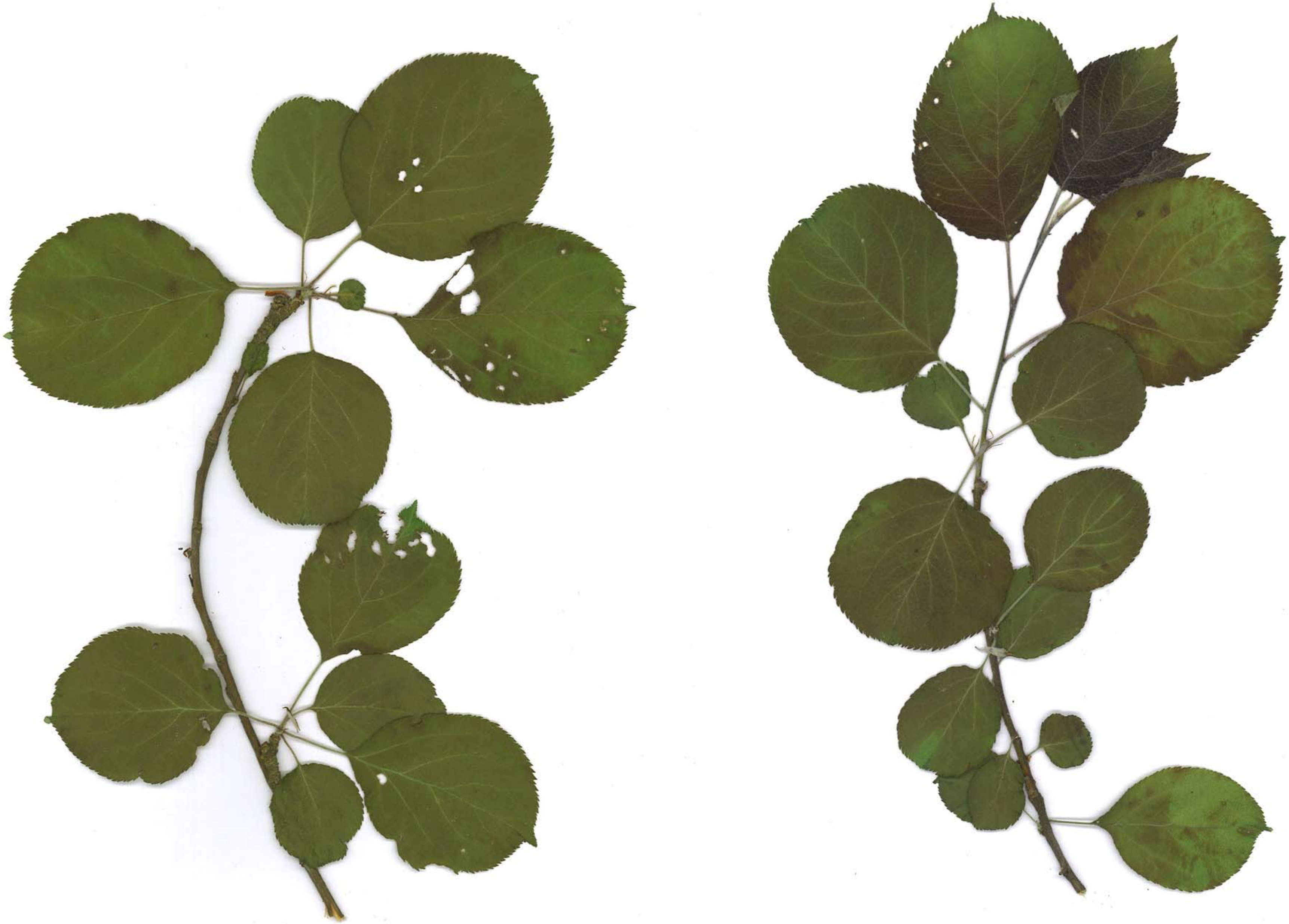

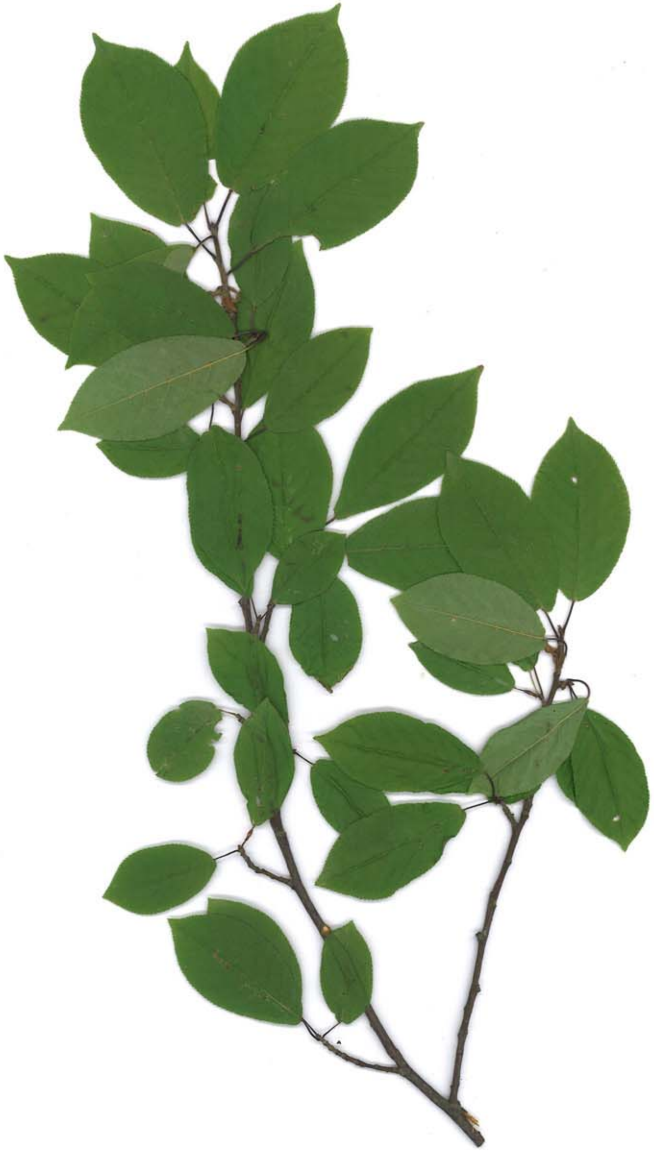

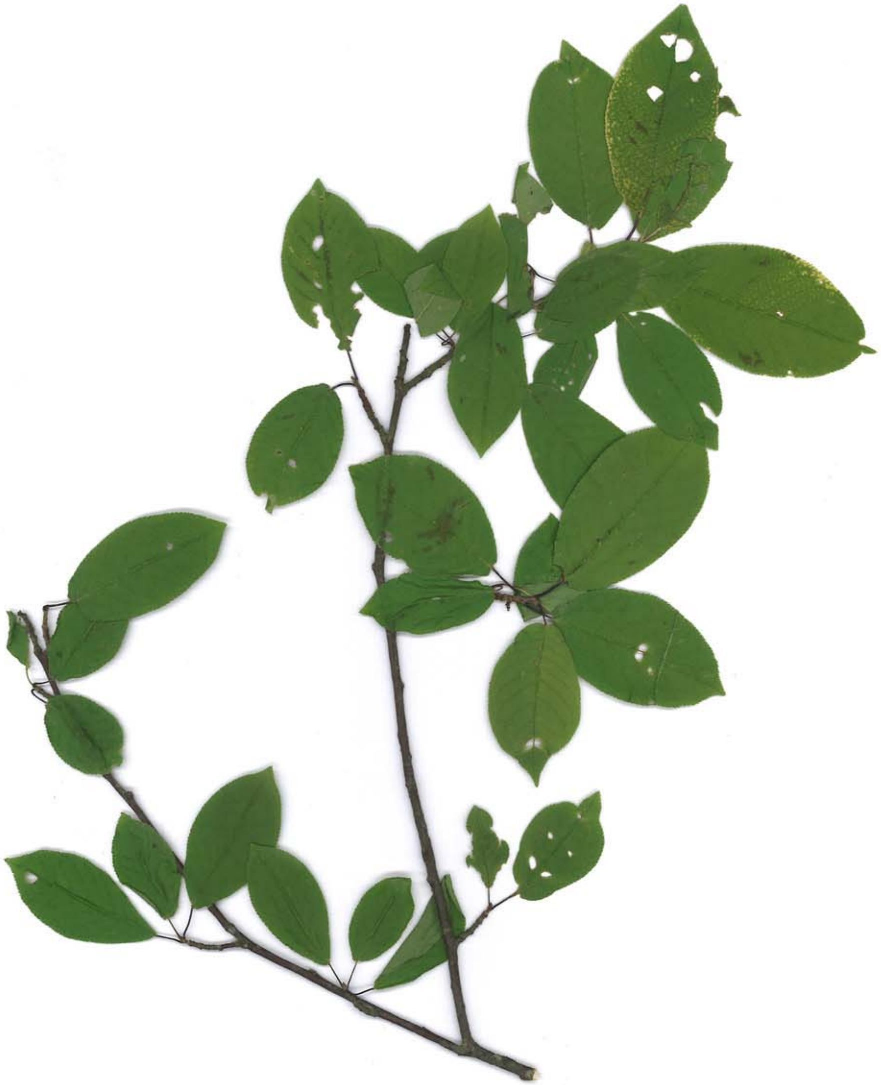

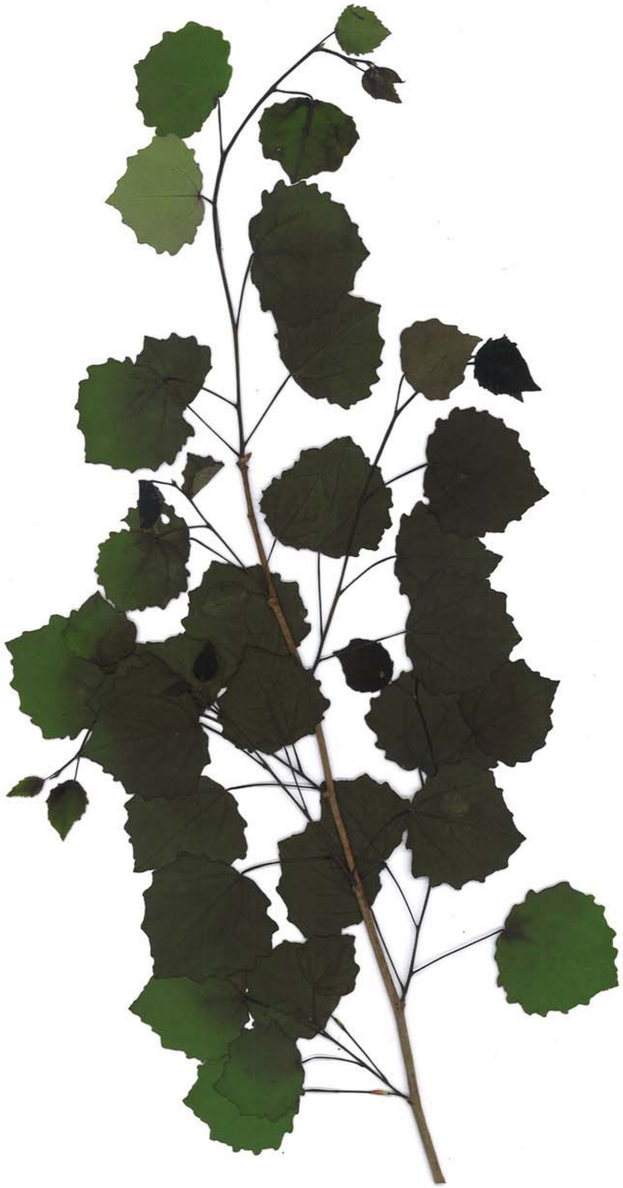

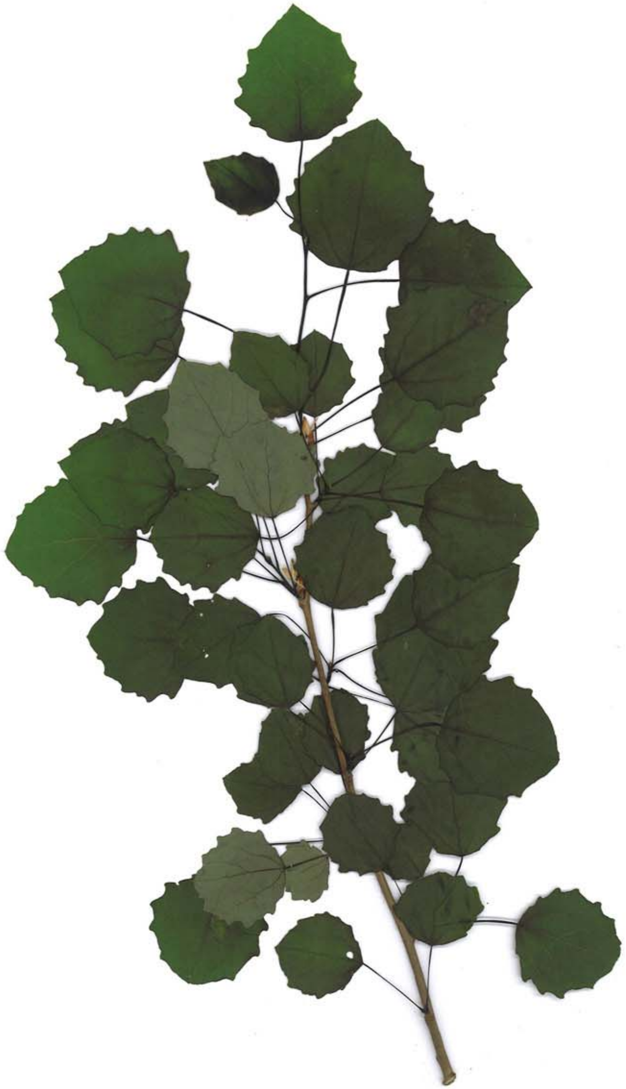

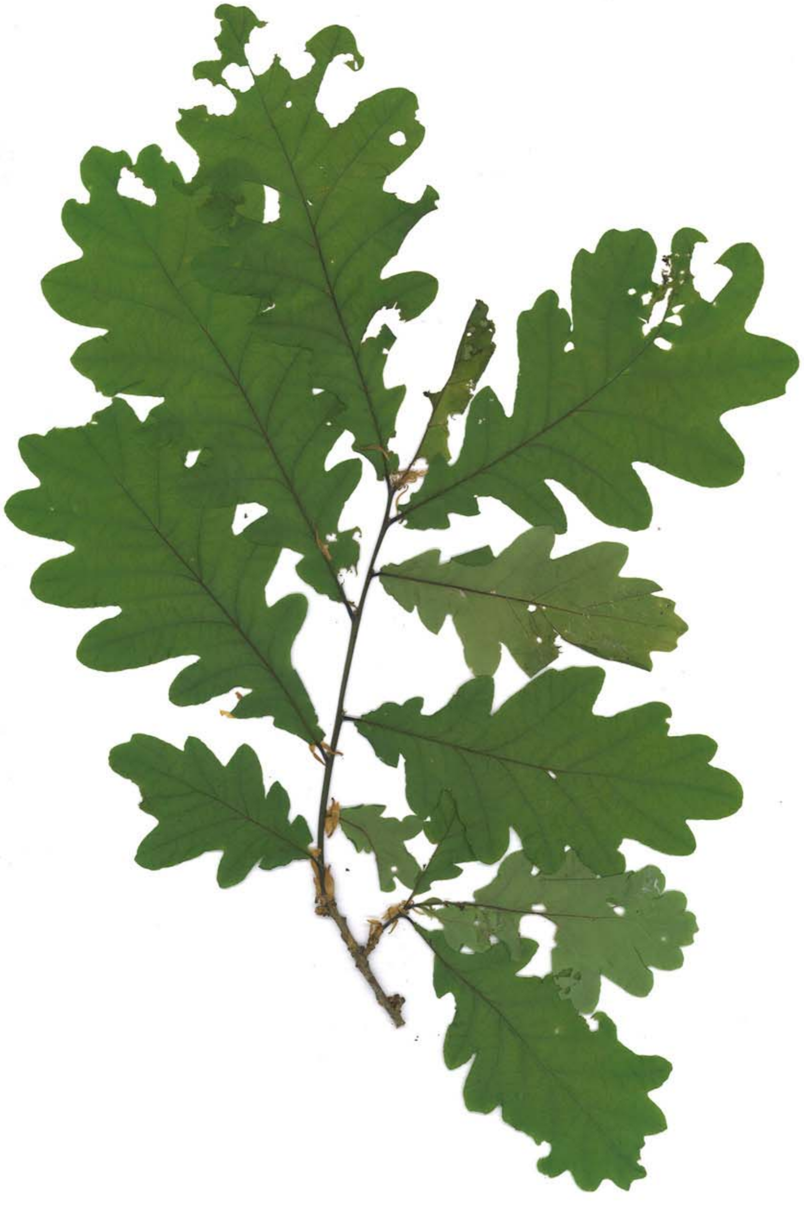
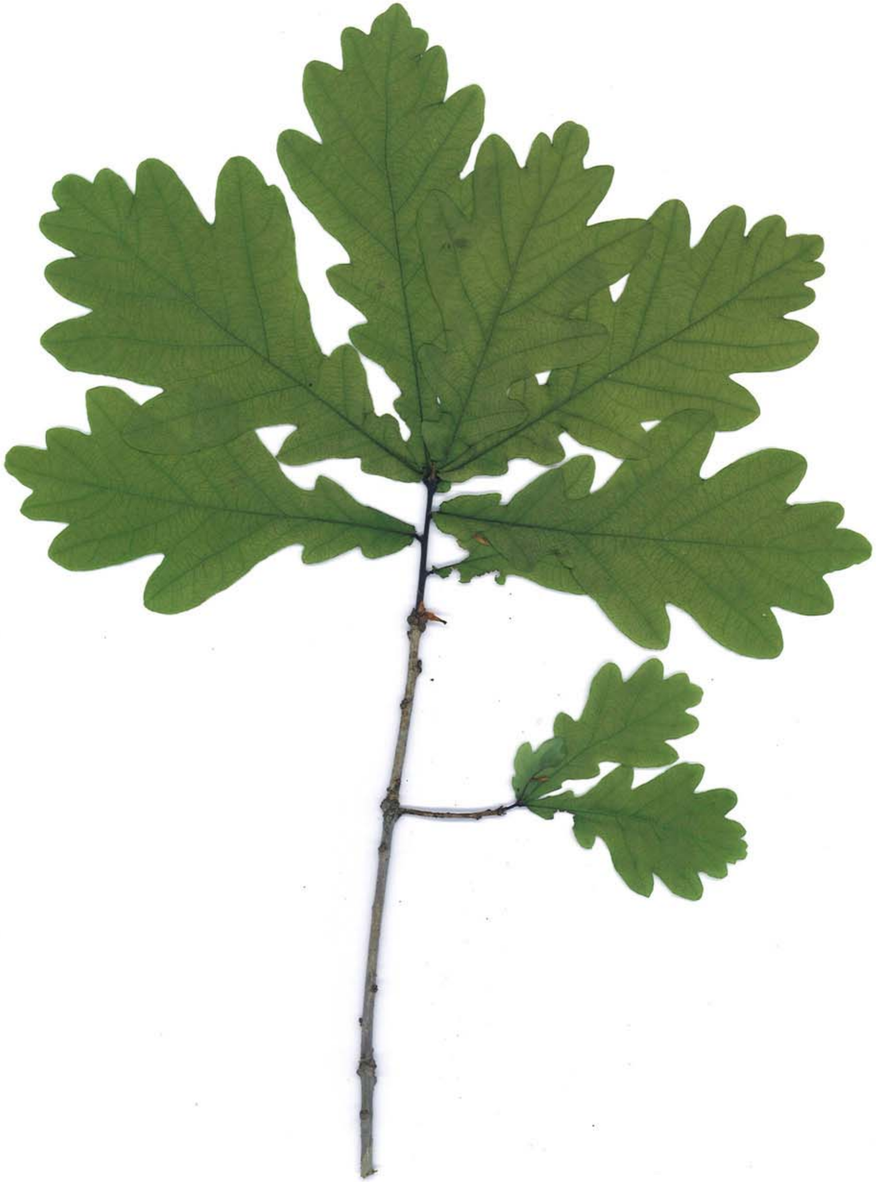

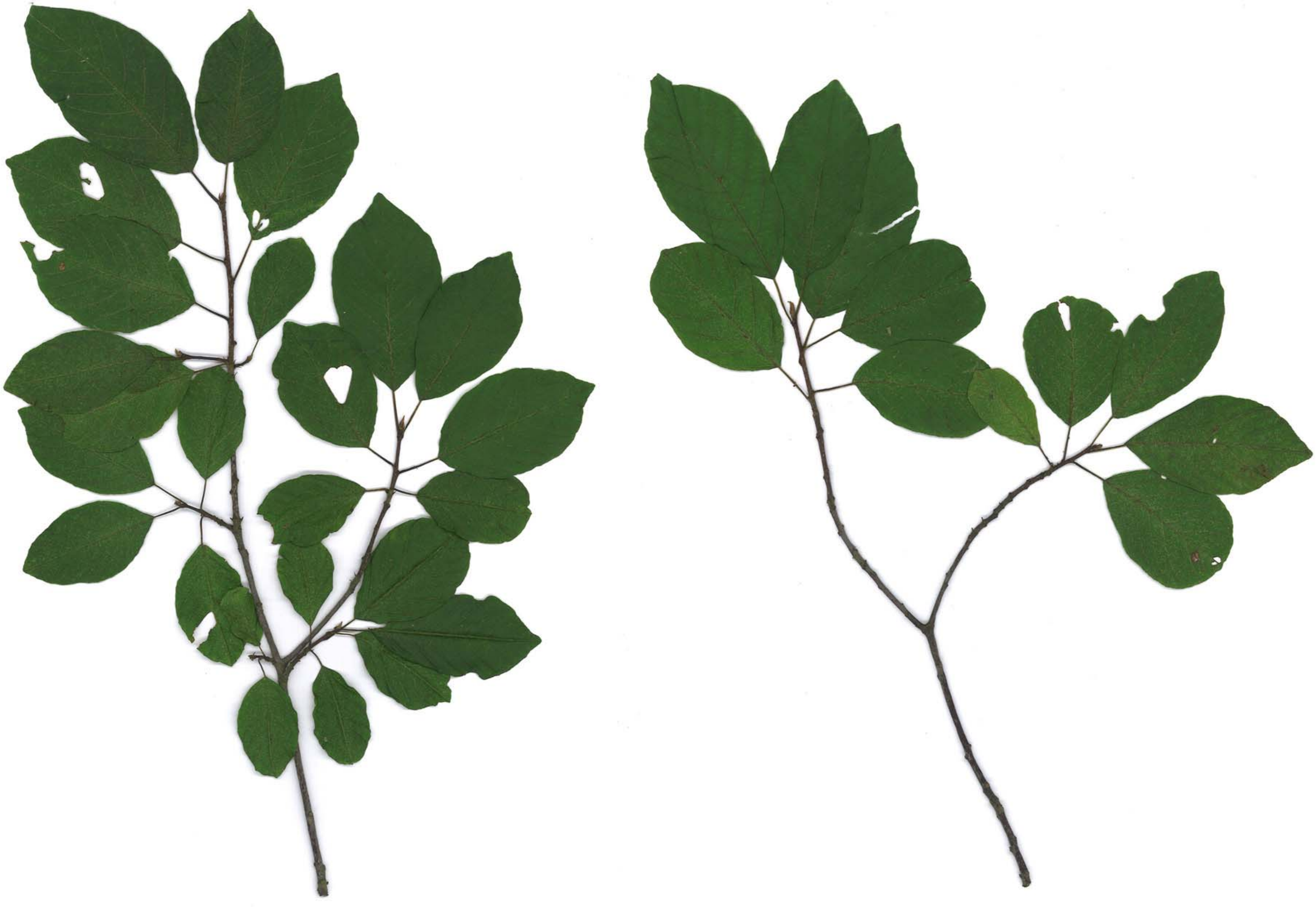

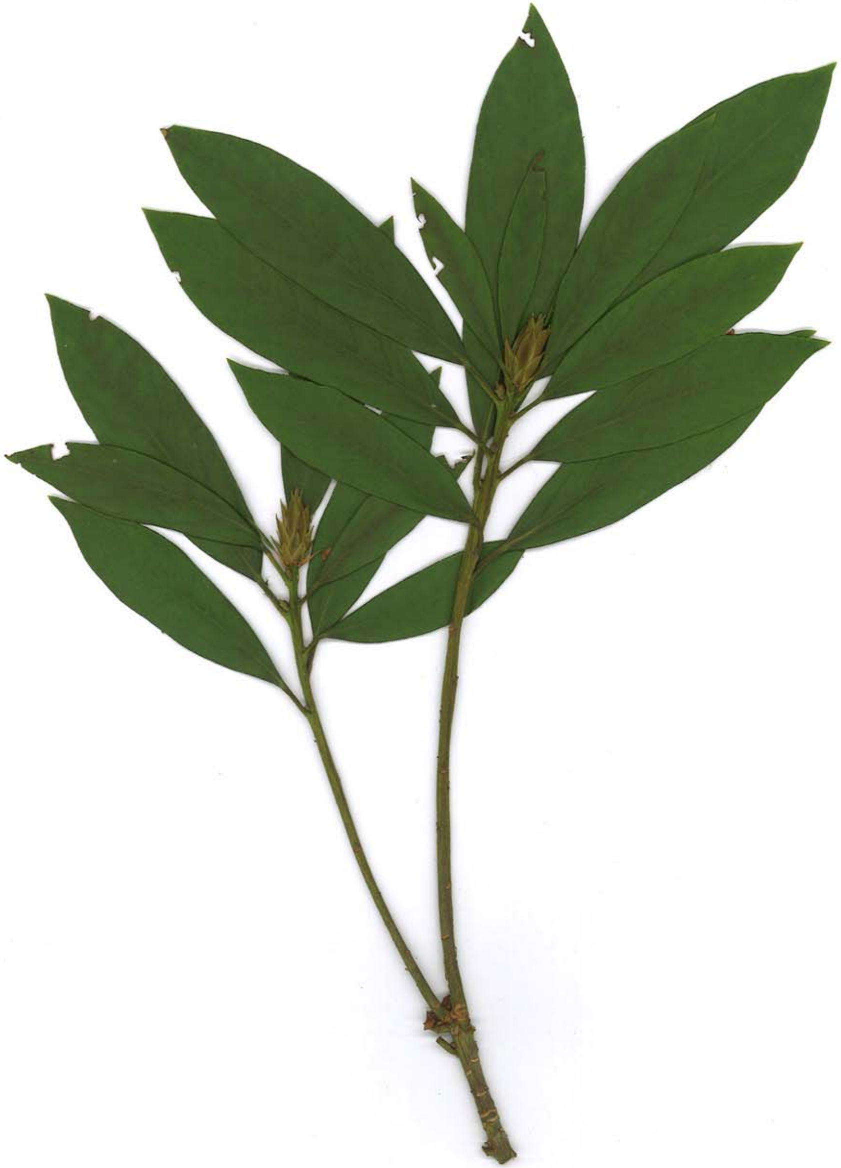

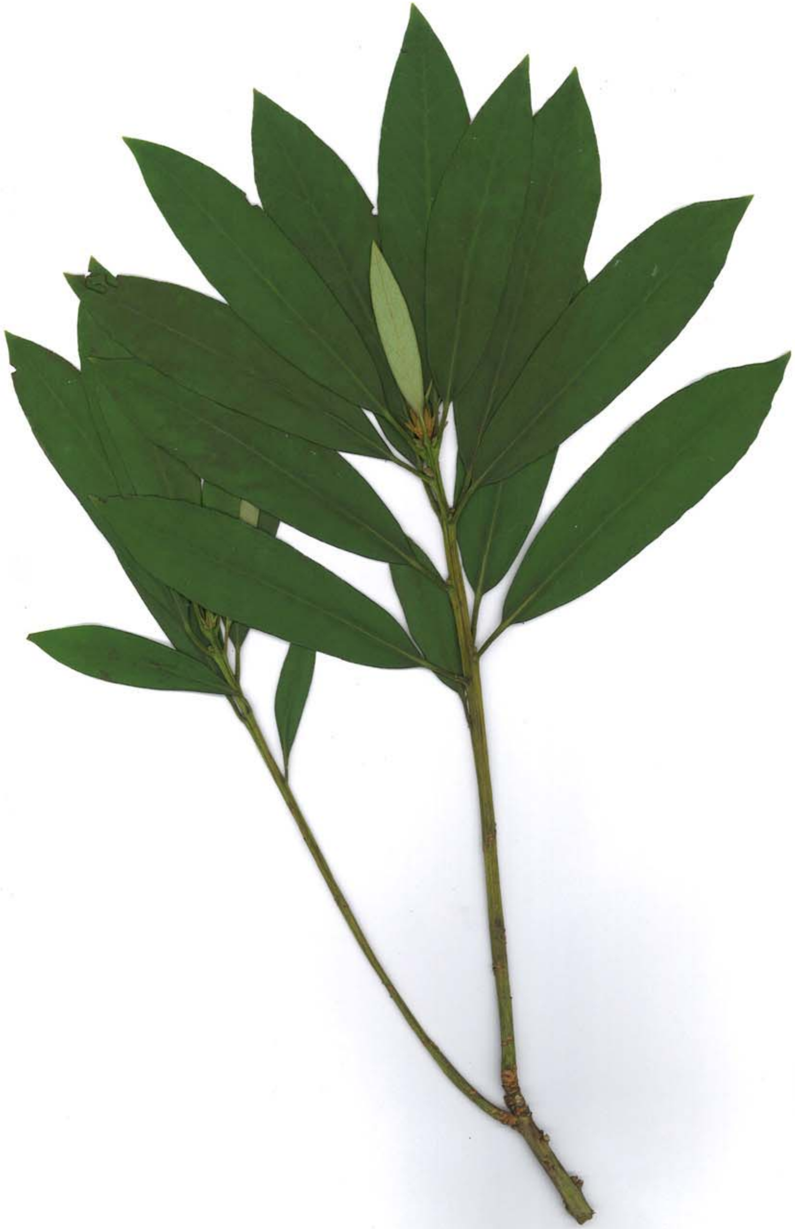

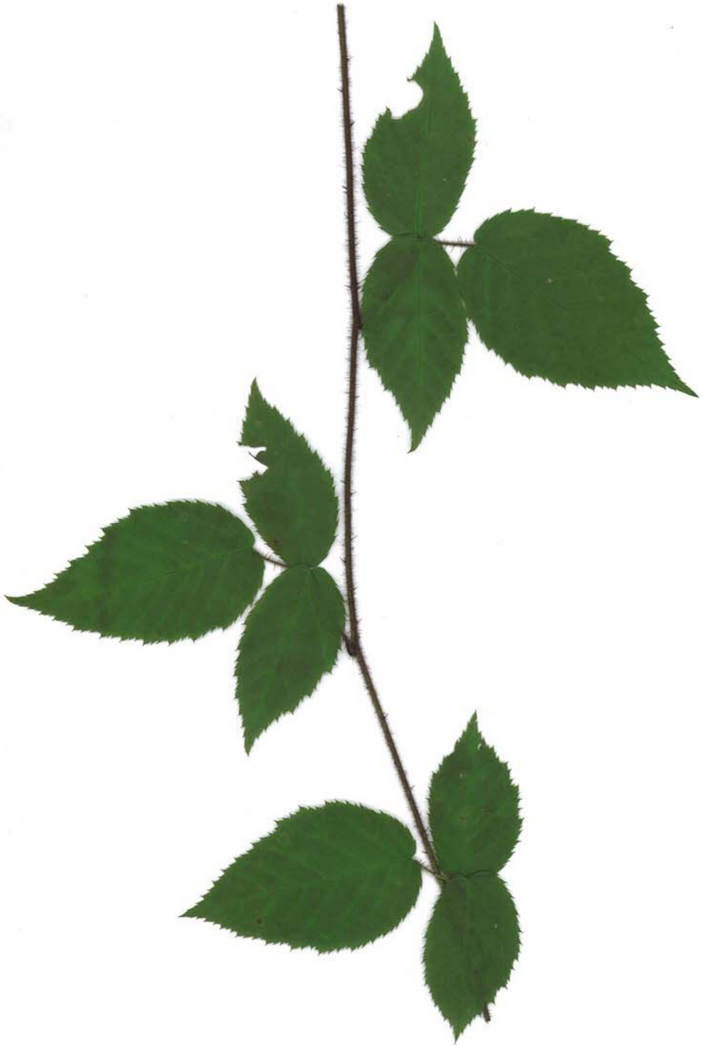

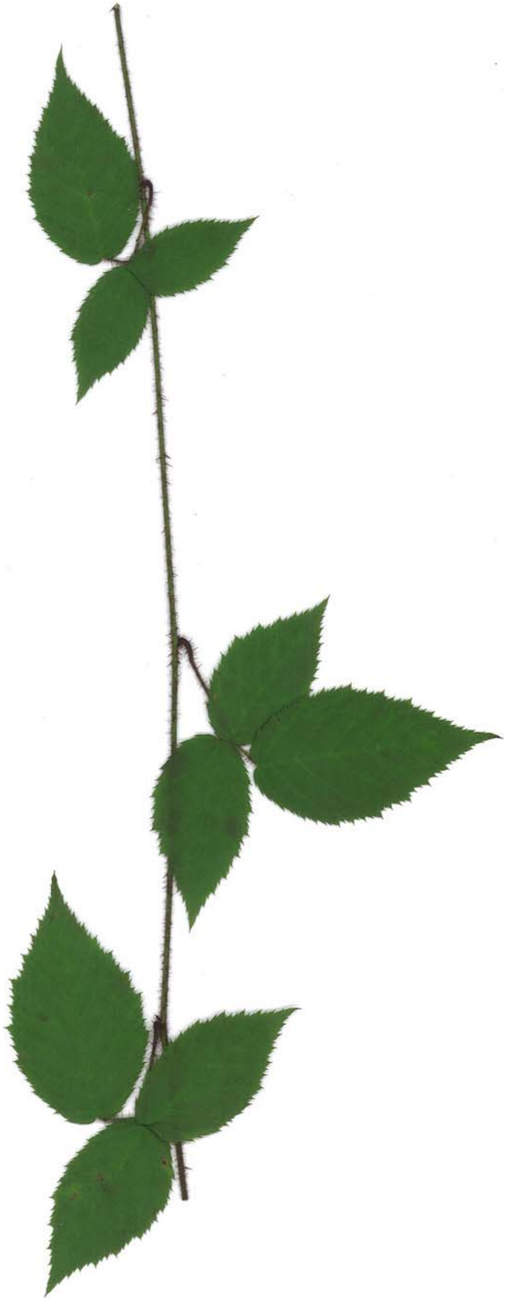

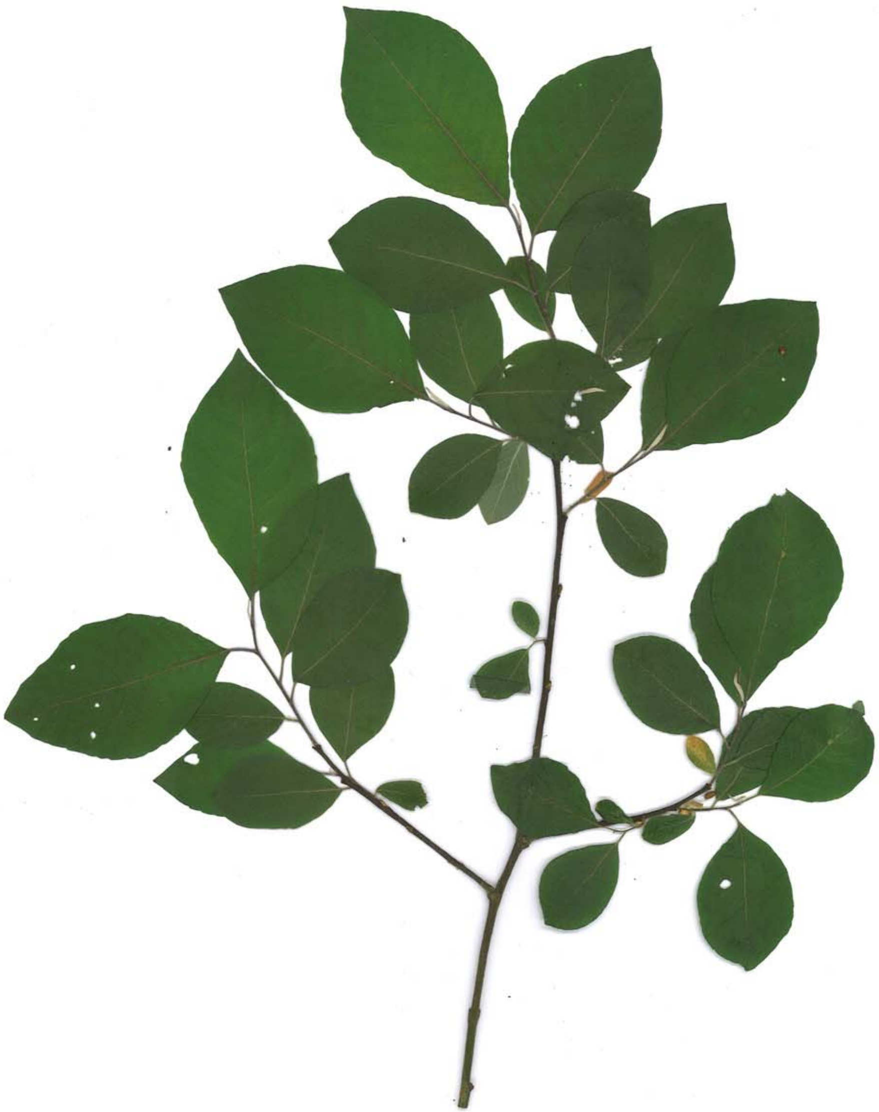
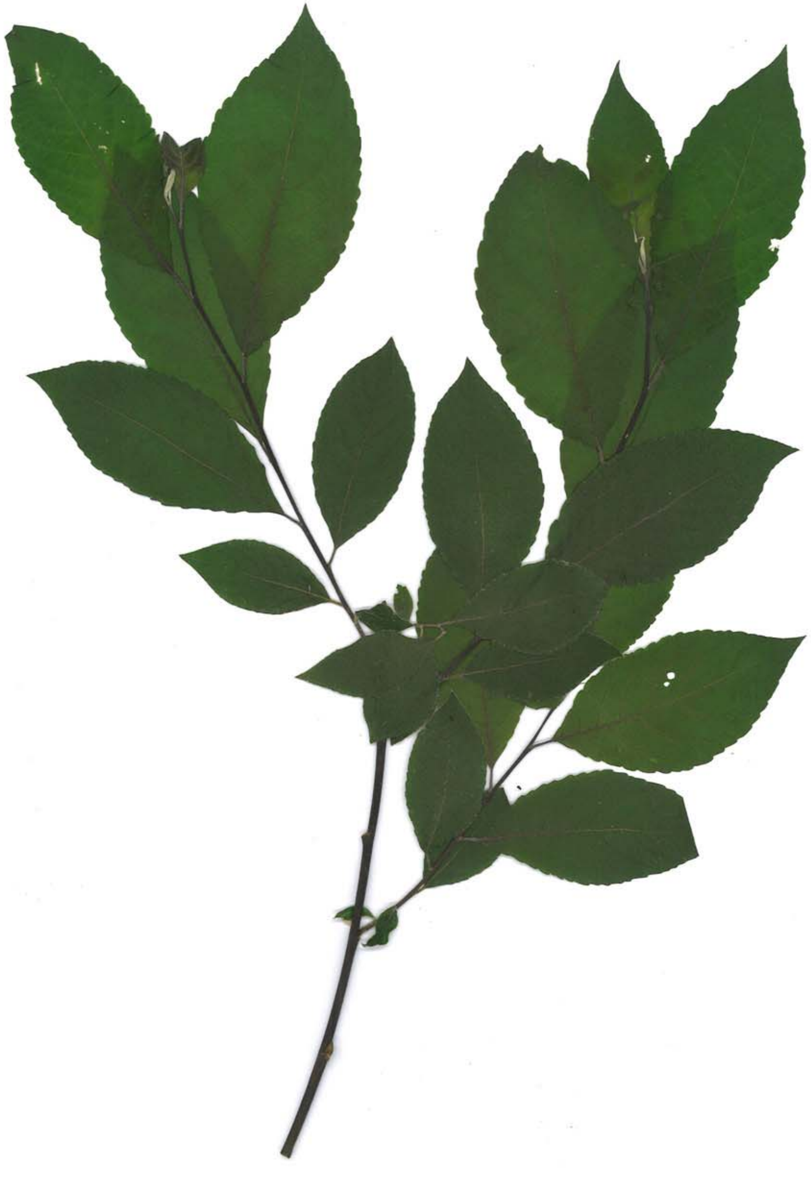

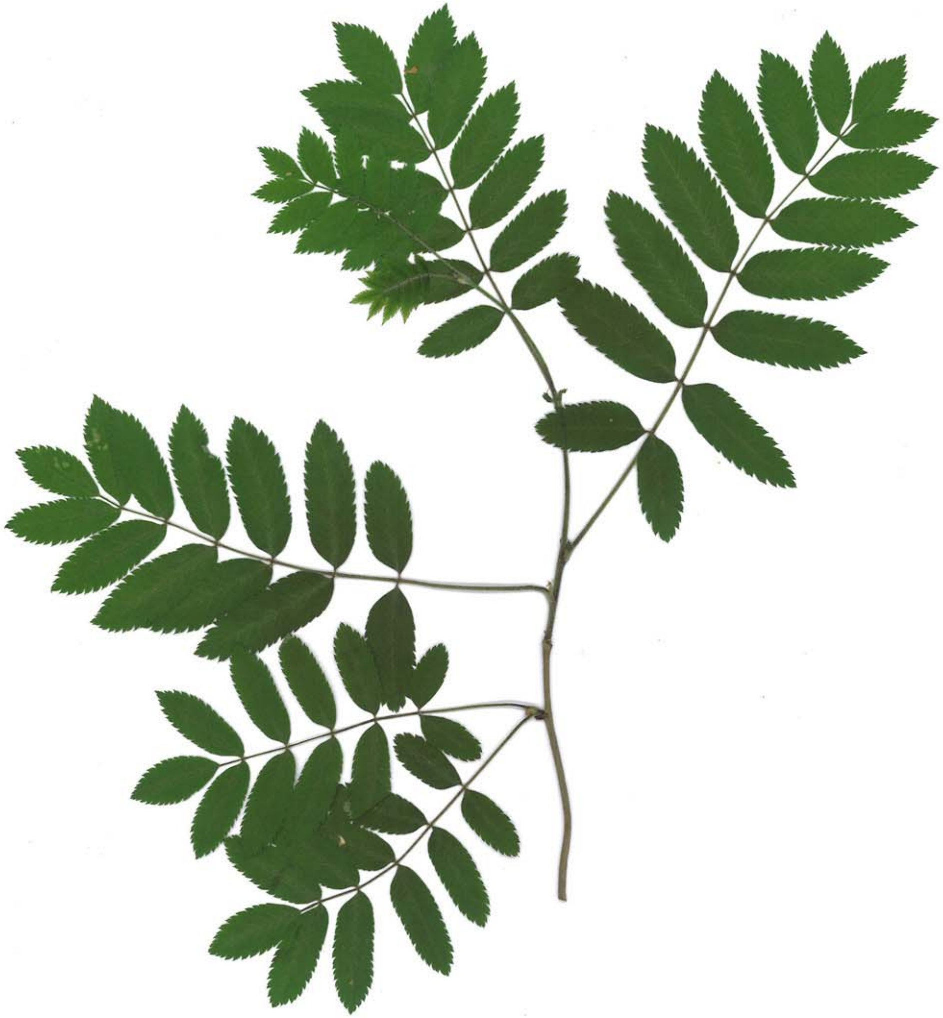
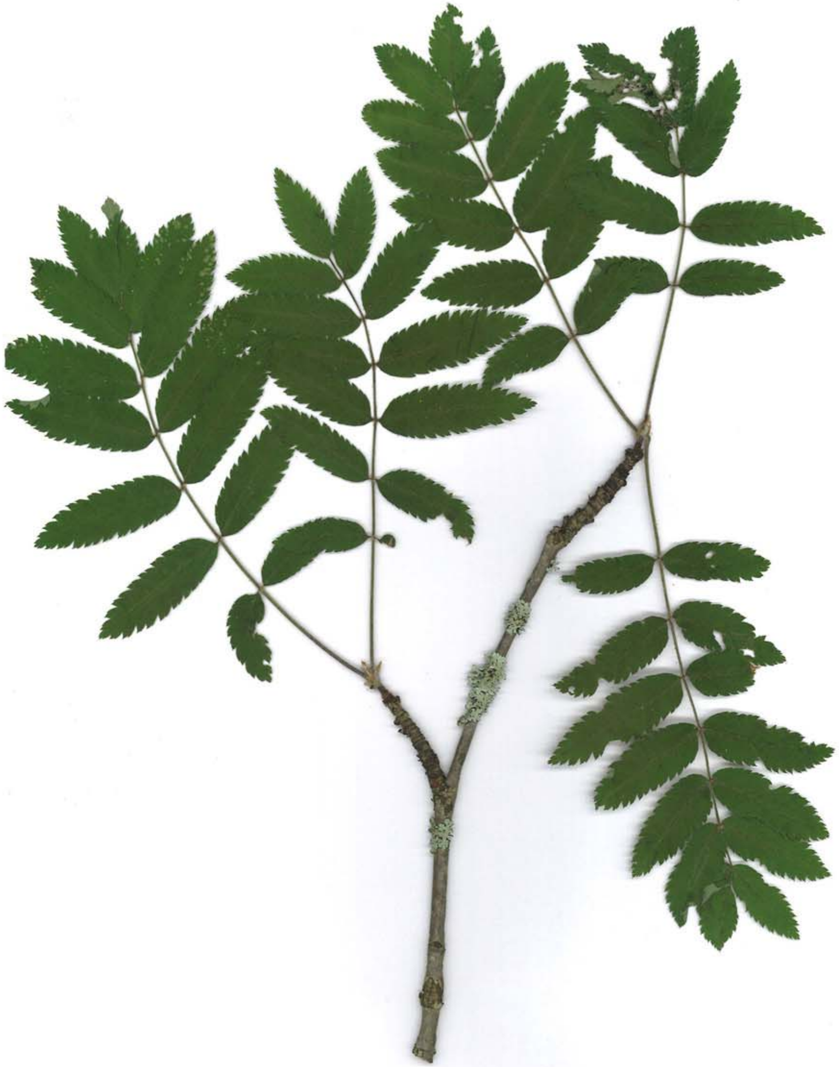

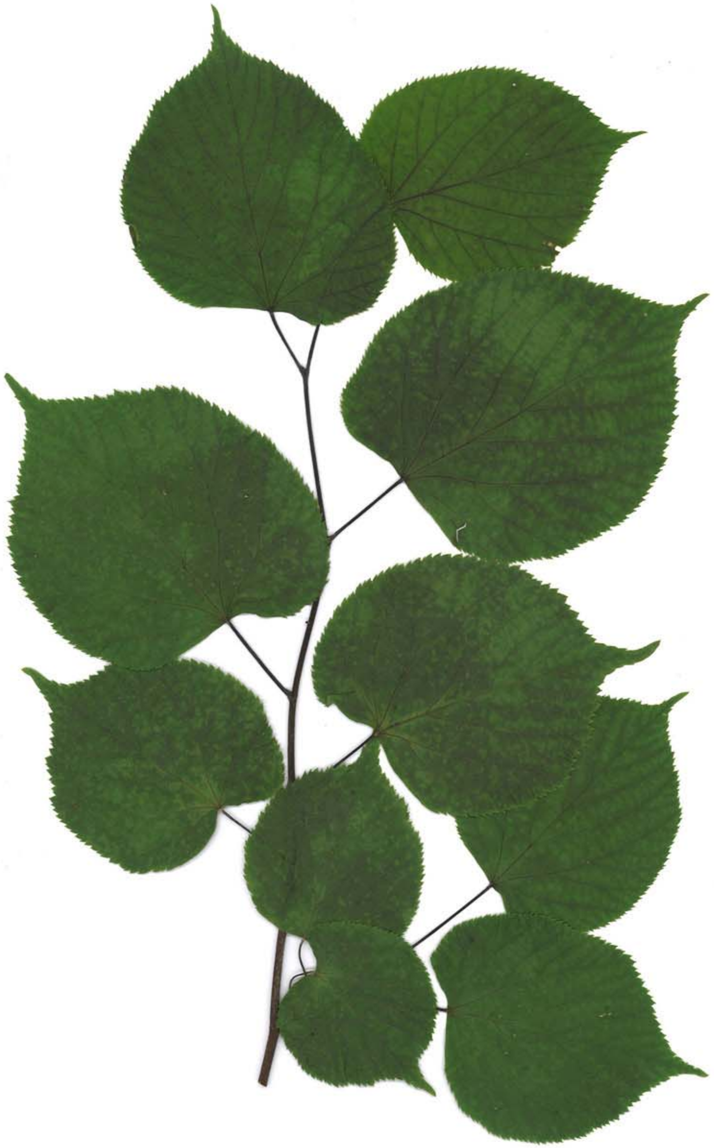

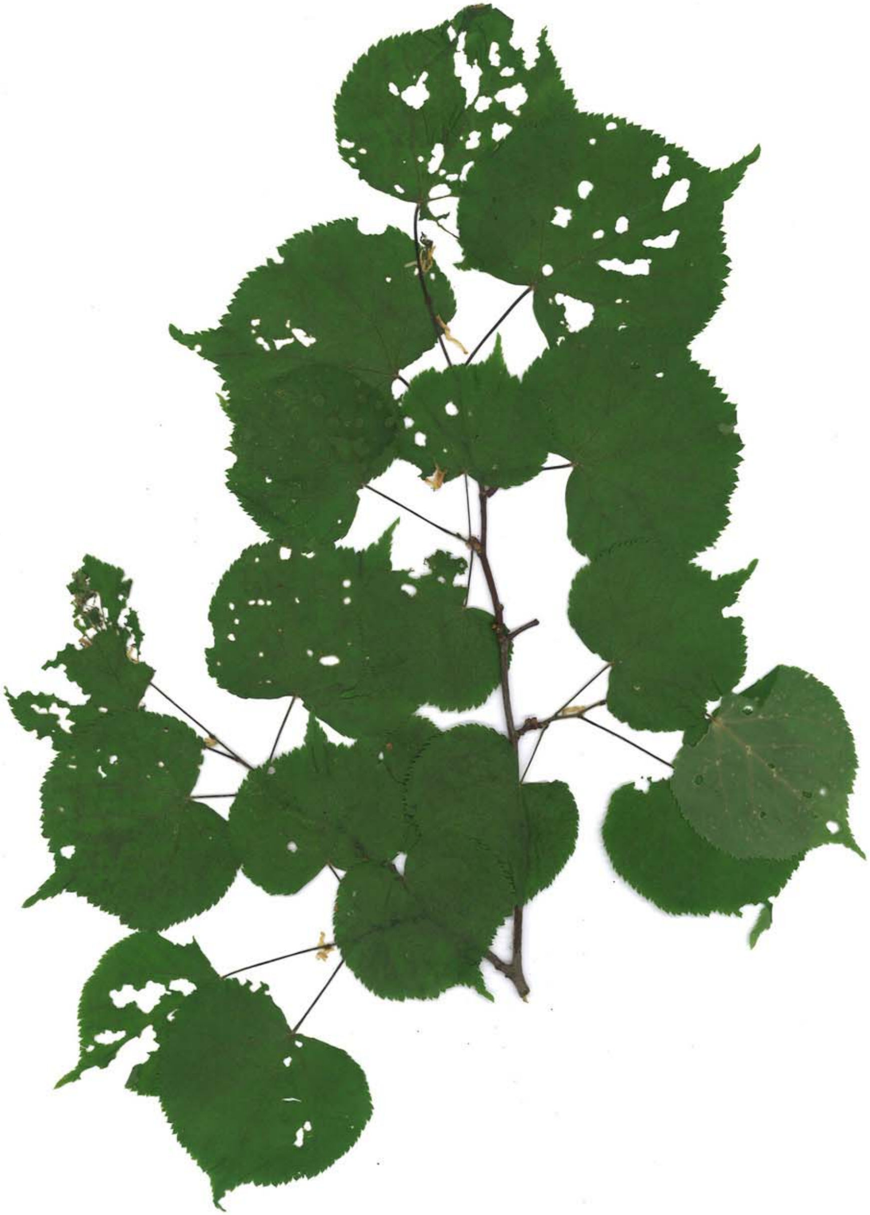

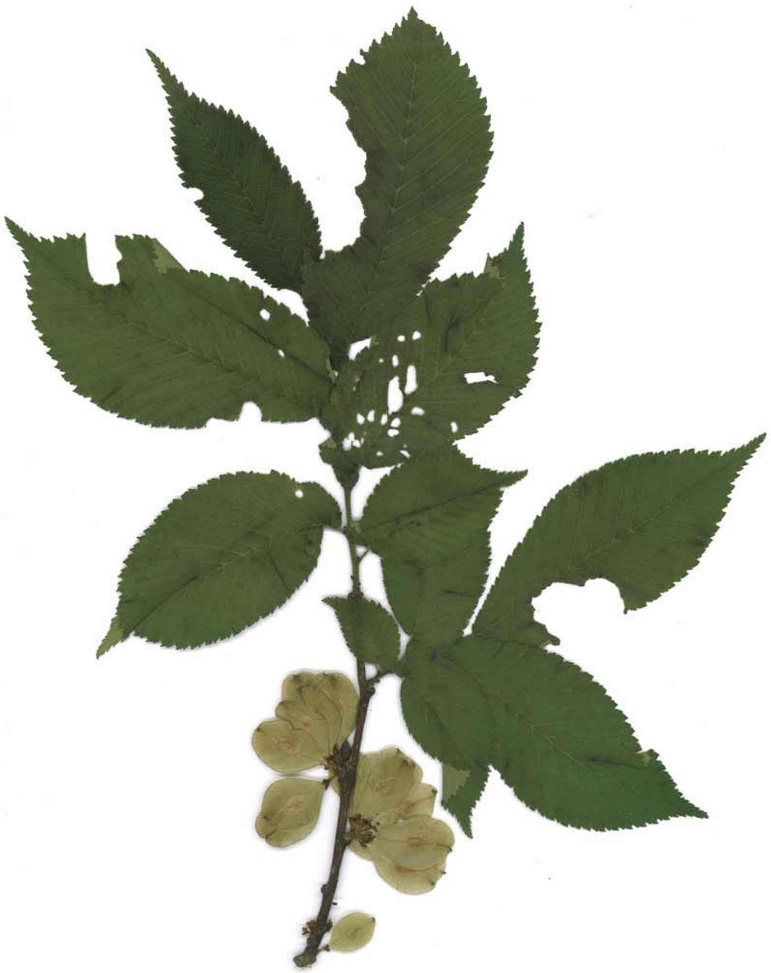

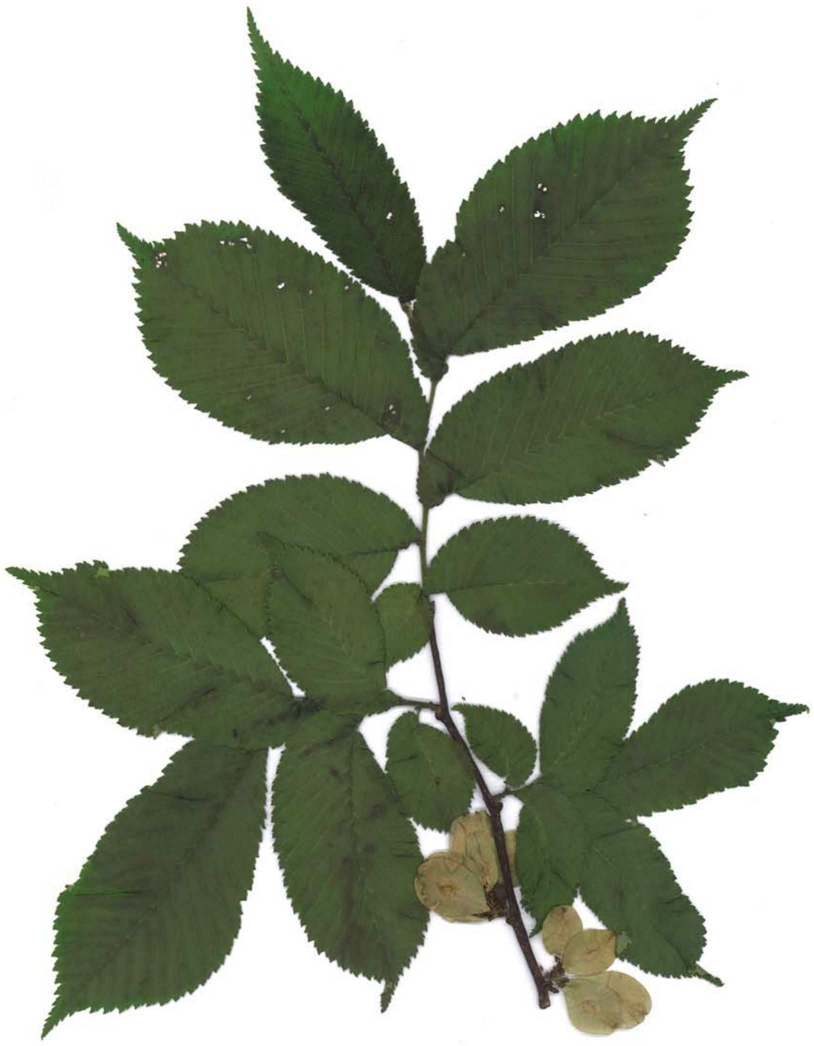

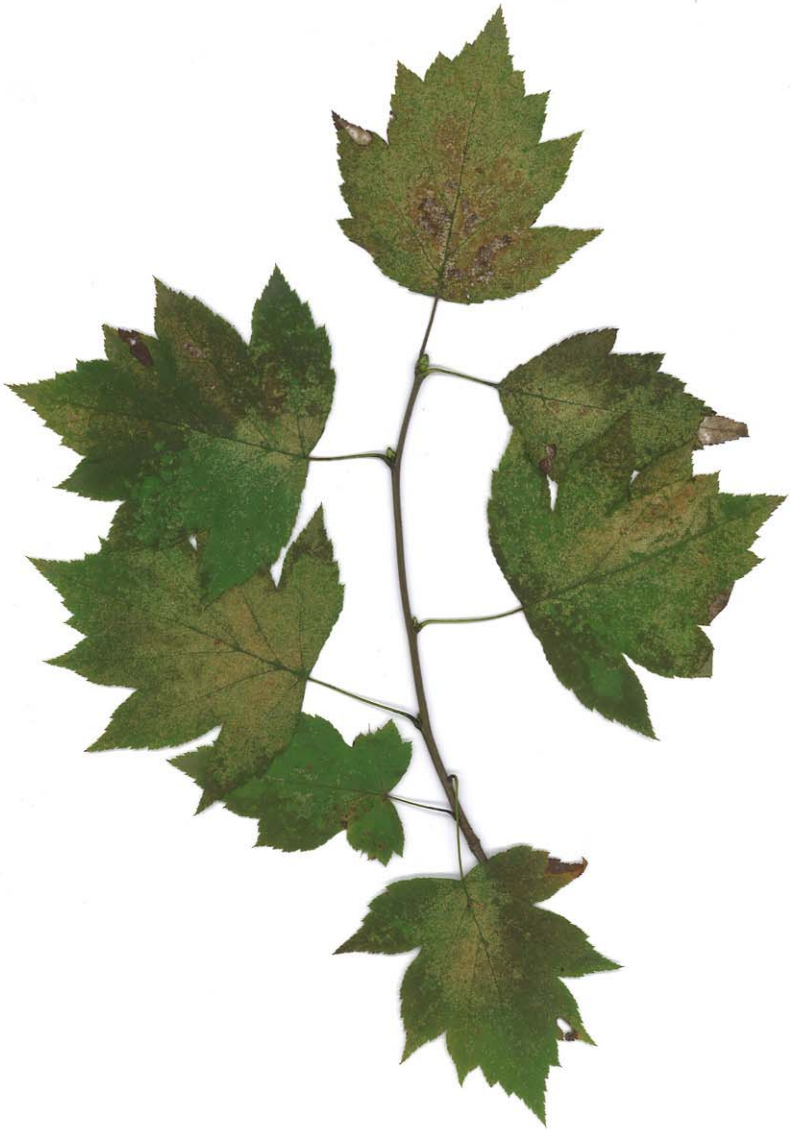
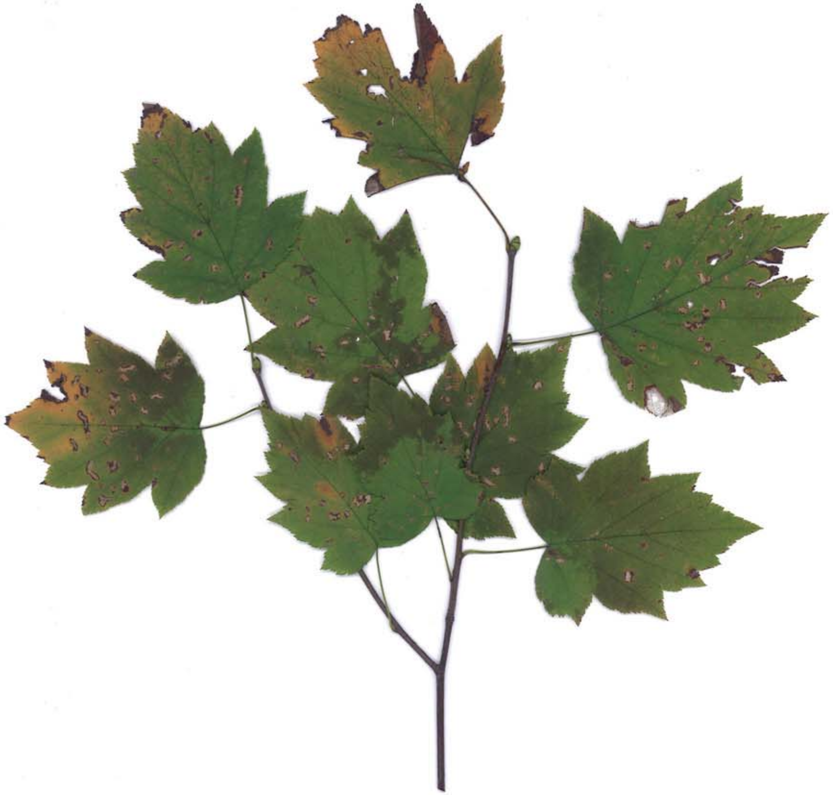

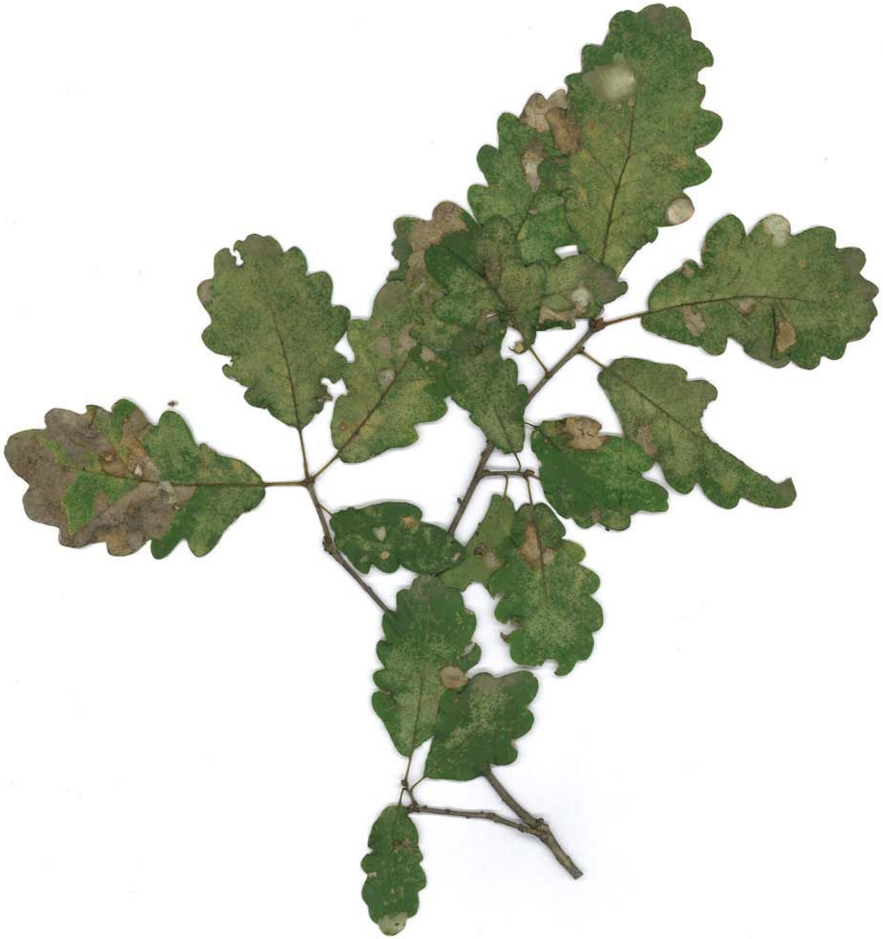

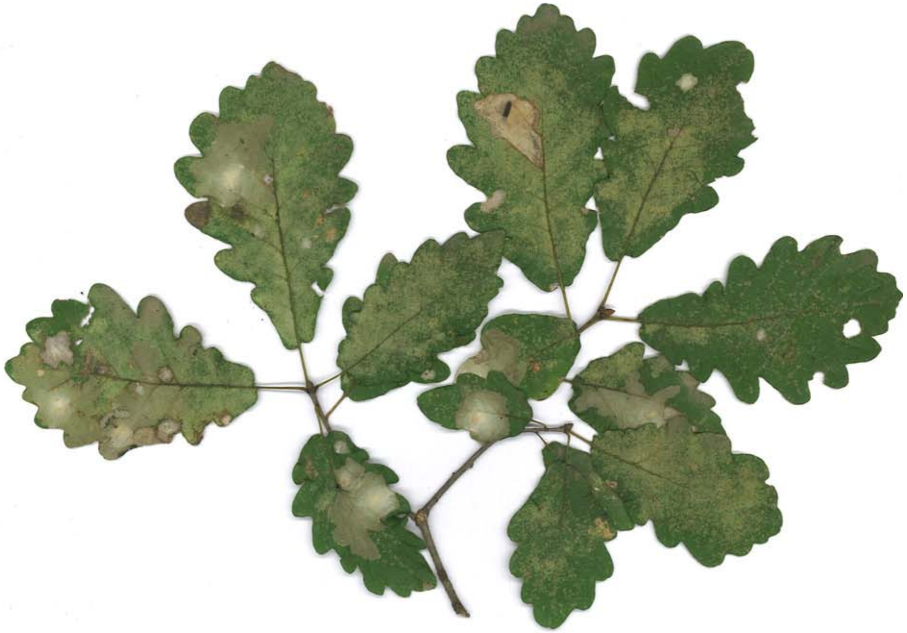

Please list the criteria you used to select between the two specimens within each pair :

- 1.
- 2.
- 3.

Please provide some information about you (by underlining or boldfacing the correct answers):

- Your experience with plant sampling for herbarium collections: less than 10 years, 10–20 years, more than 20 years
- Your experience with curation of herbarium collections: less than 10 years, 10–20 years, more than 20 years
- The number of scientific papers authored/co-authored by you: less than 10; 10–50; more than 50.
- Your age: less than 45 years; more than 45 years.

Please save this file and return it to Mikhail Kozlov (mikoz@utu.fi).

Many thanks for your time and help!

Supplementary Data S1

Losses of leaf area to insects in field collected samples (columns are space-separated).

Column 1: Sample type (ecological vs botanical).
Column 2: Locality (St_Petersburg vs Lembolovo)
Column 3: Plant name (genus_species).
Column 4: Person who collected the sample (coded ”Collector01” to ”Collector26”).
Column 5: Loss of leaf area to insects (percent).

```
botanical Lembolovo Alnus_incana . 10.2000
botanical Lembolovo Alnus_incana . 3.3182
botanical Lembolovo Alnus_incana . 3.5833
botanical Lembolovo Alnus_incana . 0.6429
botanical Lembolovo Alnus_incana . 3.6500
botanical Lembolovo Alnus_incana . 9.7500
botanical Lembolovo Alnus_incana Collector01 14.8056
botanical Lembolovo Alnus_incana . 8.8125
botanical Lembolovo Betula_pendula Collector03 1.4706
botanical Lembolovo Betula_pendula Collector06 7.5000
botanical Lembolovo Betula_pendula . 15.8000
botanical Lembolovo Betula_pendula Collector06 13.9444
botanical Lembolovo Betula_pubescens Collector03 15.0000
botanical Lembolovo Betula_pubescens . 3.2857
botanical Lembolovo Betula_pubescens . 3.3846
botanical Lembolovo Betula_pubescens Collector08 4.3889
botanical Lembolovo Betula_pubescens . 1.5556
botanical Lembolovo Betula_pubescens Collector01 20.7188
botanical Lembolovo Betula_pubescens . 14.8148
botanical Lembolovo Frangula_alnus Collector09 2.0000
botanical Lembolovo Frangula_alnus Collector01 5.0250
botanical Lembolovo Frangula_alnus . 2.0625
botanical Lembolovo Frangula_alnus Collector05 1.0714
botanical Lembolovo Frangula_alnus . 0.1429
botanical Lembolovo Frangula_alnus . 0.3333
botanical Lembolovo Frangula_alnus Collector03 0.4000
botanical Lembolovo Frangula_alnus Collector05 0.4000
botanical Lembolovo Frangula_alnus Collector01 11.4000
botanical Lembolovo Frangula_alnus Collector08 0.4286
botanical Lembolovo Frangula_alnus Collector10 0.5000
botanical Lembolovo Frangula_alnus . 5.5000
botanical Lembolovo Frangula_alnus Collector02 4.6875
botanical Lembolovo Frangula_alnus Collector07 0.7143
botanical Lembolovo Frangula_alnus Collector03 0.8333
botanical Lembolovo Frangula_alnus Collector06 2.9286
botanical Lembolovo Frangula_alnus Collector01 3.9583
botanical Lembolovo Prunus_padus . 8.0526
botanical Lembolovo Prunus_padus Collector10 6.4286
botanical Lembolovo Rubus_idaeus Collector02 3.0000
botanical Lembolovo Rubus_idaeus Collector03 0.0833
botanical Lembolovo Rubus_idaeus Collector07 0.1000
botanical Lembolovo Rubus_idaeus Collector01 0.5000
botanical Lembolovo Rubus_idaeus Collector01 3.6429
botanical Lembolovo Rubus_idaeus Collector10 3.7500
botanical Lembolovo Rubus_saxatilus Collector04 17.1000
botanical Lembolovo Rubus_saxatilus . 7.7500
botanical Lembolovo Vaccinium_myrtillus Collector08 1.0045
botanical Lembolovo Vaccinium_myrtillus Collector07 4.0077
botanical Lembolovo Vaccinium_myrtillus Collector02 5.0357
botanical Lembolovo Vaccinium_myrtillus Collector01 10.0615
```

```
botanical Lembolovo Vaccinium_myrtillus Collector10 3.7164
botanical Lembolovo Vaccinium_vitis-idaea Collector02 3.1053
botanical Lembolovo Vaccinium_vitis-idaea . 4.4474
botanical Lembolovo Vaccinium_vitis-idaea Collector01 5.5476
botanical Lembolovo Vaccinium_vitis-idaea Collector01 1.6951
botanical Lembolovo Vaccinium_vitis-idaea Collector07 7.7000
botanical Lembolovo Vaccinium_vitis-idaea Collector03 3.9130
botanical St_Petersburg Acer_platanoides Collector24 3.0000
botanical St_Petersburg Acer_platanoides Collector22 5.0000
botanical St_Petersburg Acer_platanoides Collector14 7.0000
botanical St_Petersburg Acer_platanoides Collector18 9.0000
botanical St_Petersburg Acer_platanoides Collector23 10.3125
botanical St_Petersburg Acer_platanoides Collector22 2.3750
botanical St_Petersburg Acer_platanoides Collector25 0.5000
botanical St_Petersburg Acer_platanoides Collector16 2.5000
botanical St_Petersburg Acer_platanoides Collector15 1.7500
botanical St_Petersburg Acer_platanoides Collector21 7.8000
botanical St_Petersburg Alnus_glutinosa Collector18 1.0000
botanical St_Petersburg Alnus_glutinosa Collector24 5.1111
botanical St_Petersburg Alnus_glutinosa Collector22 6.1250
botanical St_Petersburg Alnus_glutinosa Collector16 1.2857
botanical St_Petersburg Alnus_glutinosa Collector14 0.4000
botanical St_Petersburg Alnus_glutinosa Collector17 0.5294
botanical St_Petersburg Alnus_glutinosa Collector23 1.6154
botanical St_Petersburg Alnus_glutinosa Collector15 5.6250
botanical St_Petersburg Alnus_glutinosa Collector21 5.6667
botanical St_Petersburg Alnus_glutinosa Collector20 1.6765
botanical St_Petersburg Alnus_glutinosa Collector12 0.8077
botanical St_Petersburg Alnus_glutinosa Collector25 7.8333
botanical St_Petersburg Alnus_glutinosa Collector11 2.8846
botanical St_Petersburg Alnus_glutinosa Collector13 0.9750
botanical St_Petersburg Betula_pubescens Collector16 4.0714
botanical St_Petersburg Betula_pubescens Collector22 27.1618
botanical St_Petersburg Betula_pubescens Collector21 5.1765
botanical St_Petersburg Betula_pubescens Collector24 10.3667
botanical St_Petersburg Betula_pubescens Collector11 5.3750
botanical St_Petersburg Betula_pubescens Collector23 15.5192
botanical St_Petersburg Betula_pubescens Collector25 1.0500
botanical St_Petersburg Betula_pubescens Collector15 3.5357
botanical St_Petersburg Betula_pubescens Collector19 2.8500
botanical St_Petersburg Prunus_padus Collector25 6.6970
botanical St_Petersburg Prunus_padus Collector15 7.8125
botanical St_Petersburg Prunus_padus Collector21 6.8333
botanical St_Petersburg Quercus_robur Collector12 1.0000
botanical St_Petersburg Quercus_robur Collector11 5.1471
botanical St_Petersburg Quercus_robur Collector13 4.1667
botanical St_Petersburg Quercus_robur Collector18 10.2000
botanical St_Petersburg Quercus_robur Collector24 4.2833
botanical St_Petersburg Quercus_robur Collector14 0.4375
botanical St_Petersburg Quercus_robur Collector16 1.5714
botanical St_Petersburg Quercus_robur Collector20 5.6154
botanical St_Petersburg Quercus_robur Collector23 3.8000
botanical St_Petersburg Quercus_robur Collector22 5.8500
botanical St_Petersburg Quercus_robur Collector17 0.8571
botanical St_Petersburg Rubus_idaeus Collector11 1.0000
botanical St_Petersburg Rubus_idaeus Collector18 2.0000
botanical St_Petersburg Rubus_idaeus Collector14 1.1667
botanical St_Petersburg Rubus_idaeus Collector13 3.2414
botanical St_Petersburg Rubus_idaeus Collector17 3.3333
botanical St_Petersburg Rubus_idaeus Collector25 4.3621
botanical St_Petersburg Rubus_idaeus Collector24 8.5000
```

```
botanical St_Petersburg Rubus_idaeus Collector15 4.5577
botanical St_Petersburg Rubus_idaeus Collector22 1.6923
botanical St_Petersburg Rubus_idaeus Collector23 0.7500
botanical St_Petersburg Rubus_idaeus Collector21 4.7727
botanical St_Petersburg Rubus_idaeus Collector20 3.8333
botanical St_Petersburg Rubus_idaeus Collector16 3.8750
botanical St_Petersburg Rubus_idaeus Collector12 1.9000
botanical St_Petersburg Salix_fragilis Collector11 5.1071
botanical St_Petersburg Salix_fragilis Collector24 4.1667
botanical St_Petersburg Salix_fragilis Collector16 4.1875
botanical St_Petersburg Salix_fragilis Collector22 3.2308
botanical St_Petersburg Salix_fragilis Collector13 0.6667
botanical St_Petersburg Salix_fragilis Collector14 1.6818
botanical St_Petersburg Salix_fragilis Collector17 0.7500
botanical St_Petersburg Salix_fragilis Collector12 0.8333
botanical St_Petersburg Salix_fragilis Collector18 0.9286
botanical St_Petersburg Salix_fragilis Collector20 2.9545
botanical St_Petersburg Salix_fragilis Collector23 2.9688
botanical St_Petersburg Sorbus_aucuparia Collector12 9.0000
botanical St_Petersburg Sorbus_aucuparia Collector17 9.0000
botanical St_Petersburg Sorbus_aucuparia Collector20 1.4286
botanical St_Petersburg Sorbus_aucuparia Collector13 3.8000
botanical St_Petersburg Tilia_cordata Collector11 4.0000
botanical St_Petersburg Tilia_cordata Collector16 13.0000
botanical St_Petersburg Tilia_cordata Collector25 6.1000
botanical St_Petersburg Tilia_cordata Collector13 8.1389
botanical St_Petersburg Tilia_cordata Collector20 6.1786
botanical St_Petersburg Tilia_cordata Collector23 17.4286
botanical St_Petersburg Tilia_cordata Collector14 0.5000
botanical St_Petersburg Tilia_cordata Collector15 5.5000
botanical St_Petersburg Tilia_cordata Collector12 10.5417
botanical St_Petersburg Tilia_cordata Collector22 2.7222
botanical St_Petersburg Tilia_cordata Collector17 7.7778
botanical St_Petersburg Tilia_cordata Collector24 7.8750
botanical St_Petersburg Tilia_cordata Collector18 3.9000
botanical St_Petersburg Tilia_cordata Collector21 11.9762
botanical St_Petersburg Ulmus_glabra Collector14 1.0000
botanical St_Petersburg Ulmus_glabra Collector21 3.0000
botanical St_Petersburg Ulmus_glabra Collector25 7.2000
botanical St_Petersburg Ulmus_glabra Collector22 16.2500
botanical St_Petersburg Ulmus_glabra Collector18 1.3333
botanical St_Petersburg Ulmus_glabra Collector17 0.3529
botanical St_Petersburg Ulmus_glabra Collector13 0.5000
botanical St_Petersburg Ulmus_glabra Collector12 2.5000
botanical St_Petersburg Ulmus_glabra Collector15 3.5000
botanical St_Petersburg Ulmus_glabra Collector11 7.6667
botanical St_Petersburg Ulmus_glabra Collector23 7.6667
botanical St_Petersburg Ulmus_glabra Collector20 0.8000
botanical St_Petersburg Ulmus_glabra Collector24 4.8571
botanical St_Petersburg Ulmus_glabra Collector16 10.8571
ecological Lembolovo Alnus_incana Collector26 13.4694
ecological Lembolovo Alnus_incana Collector26 22.5500
ecological Lembolovo Alnus_incana Collector26 3.8750
ecological Lembolovo Alnus_incana Collector26 17.8980
ecological Lembolovo Alnus_incana Collector26 16.9528
ecological Lembolovo Betula_pendula Collector26 6.1583
ecological Lembolovo Betula_pendula Collector26 7.4837
ecological Lembolovo Betula_pendula Collector26 21.6354
ecological Lembolovo Betula_pendula Collector26 15.7295
ecological Lembolovo Betula_pendula Collector26 10.9630
ecological Lembolovo Betula_pubescens Collector26 14.0521
```

```
ecological Lembolovo Betula_pubescens Collector26 16.0942
ecological Lembolovo Betula_pubescens Collector26 10.1000
ecological Lembolovo Betula_pubescens Collector26 10.2222
ecological Lembolovo Betula_pubescens Collector26 7.6455
ecological Lembolovo Frangula_alnus Collector26 2.0696
ecological Lembolovo Frangula_alnus Collector26 4.2444
ecological Lembolovo Frangula_alnus Collector26 1.4467
ecological Lembolovo Frangula_alnus Collector26 9.5159
ecological Lembolovo Frangula_alnus Collector26 3.6212
ecological Lembolovo Prunus_padus Collector26 11.0000
ecological Lembolovo Prunus_padus Collector26 11.1667
ecological Lembolovo Prunus_padus Collector26 10.8333
ecological Lembolovo Prunus_padus Collector26 9.8828
ecological Lembolovo Prunus_padus Collector26 23.9286
ecological Lembolovo Rubus_idaeus Collector26 2.0294
ecological Lembolovo Rubus_idaeus Collector26 1.0357
ecological Lembolovo Rubus_idaeus Collector26 2.2857
ecological Lembolovo Rubus_idaeus Collector26 0.9000
ecological Lembolovo Rubus_idaeus Collector26 0.9524
ecological Lembolovo Rubus_saxatilus Collector26 5.3750
ecological Lembolovo Rubus_saxatilus Collector26 3.5000
ecological Lembolovo Rubus_saxatilus Collector26 9.5294
ecological Lembolovo Rubus_saxatilus Collector26 9.6667
ecological Lembolovo Rubus_saxatilus Collector26 5.8333
ecological Lembolovo Vaccinium_myrtillus Collector26 8.1061
ecological Lembolovo Vaccinium_myrtillus Collector26 20.3788
ecological Lembolovo Vaccinium_myrtillus Collector26 6.6742
ecological Lembolovo Vaccinium_myrtillus Collector26 11.7644
ecological Lembolovo Vaccinium_myrtillus Collector26 18.9762
ecological Lembolovo Vaccinium_vitis-idaea Collector26 5.0744
ecological Lembolovo Vaccinium_vitis-idaea Collector26 9.4739
ecological Lembolovo Vaccinium_vitis-idaea Collector26 12.5400
ecological Lembolovo Vaccinium_vitis-idaea Collector26 3.5976
ecological Lembolovo Vaccinium_vitis-idaea Collector26 6.8582
ecological St_Petersburg Acer_platanoides Collector26 6.1250
ecological St_Petersburg Acer_platanoides Collector26 4.2794
ecological St_Petersburg Acer_platanoides Collector26 5.4722
ecological St_Petersburg Acer_platanoides Collector26 4.6667
ecological St_Petersburg Acer_platanoides Collector26 5.9868
ecological St_Petersburg Alnus_glutinosa Collector26 6.4737
ecological St_Petersburg Alnus_glutinosa Collector26 0.7564
ecological St_Petersburg Betula_pubescens Collector26 7.3804
ecological St_Petersburg Betula_pubescens Collector26 4.4636
ecological St_Petersburg Betula_pubescens Collector26 3.4758
ecological St_Petersburg Betula_pubescens Collector26 12.9545
ecological St_Petersburg Betula_pubescens Collector26 1.9884
ecological St_Petersburg Betula_pubescens Collector26 3.0556
ecological St_Petersburg Prunus_padus Collector26 5.1574
ecological St_Petersburg Prunus_padus Collector26 11.2297
ecological St_Petersburg Prunus_padus Collector26 3.3953
ecological St_Petersburg Prunus_padus Collector26 10.5455
ecological St_Petersburg Prunus_padus Collector26 3.9273
ecological St_Petersburg Quercus_robur Collector26 2.1889
ecological St_Petersburg Quercus_robur Collector26 7.2500
ecological St_Petersburg Quercus_robur Collector26 13.5583
ecological St_Petersburg Quercus_robur Collector26 4.8261
ecological St_Petersburg Quercus_robur Collector26 7.9787
ecological St_Petersburg Rubus_idaeus Collector26 2.0000
ecological St_Petersburg Rubus_idaeus Collector26 2.2692
ecological St_Petersburg Rubus_idaeus Collector26 1.7500
ecological St_Petersburg Rubus_idaeus Collector26 1.8333
```

```
ecological St_Petersburg Rubus_idaeus Collector26 2.9583
ecological St_Petersburg Salix_fragilis Collector26 4.0349
ecological St_Petersburg Salix_fragilis Collector26 4.1538
ecological St_Petersburg Sorbus_aucuparia Collector26 20.0455
ecological St_Petersburg Sorbus_aucuparia Collector26 24.1000
ecological St_Petersburg Tilia_cordata Collector26 12.1250
ecological St_Petersburg Tilia_cordata Collector26 8.2407
ecological St_Petersburg Tilia_cordata Collector26 9.5161
ecological St_Petersburg Tilia_cordata Collector26 11.6000
ecological St_Petersburg Tilia_cordata Collector26 13.9388
ecological St_Petersburg Ulmus_glabra Collector26 3.0556
ecological St_Petersburg Ulmus_glabra Collector26 4.0606
ecological St_Petersburg Ulmus_glabra Collector26 7.4474
ecological St_Petersburg Ulmus_glabra Collector26 18.6692
ecological St_Petersburg Ulmus_glabra Collector26 8.9574
```

Supplementary Data S2

Results of selection of plant images by staff members of different herbaria (columns are space-separated).

Column 1: Person who made the choice (coded ”Observer01” to ”Observer15”).
Column 2: Plant genus (when two species of the same genus wer included, then the second species is coded as ”plant_genus2).
Column 3: Position of the less damaged specimen in a pair (lefr or right).
Column 4: Loss of leaf area to insects in the more damaged specimen in a pair (percent).
Column 5: The absolute difference in losses of leaf area to insects in more and less damaged specimens in a pair (percent).
Column 6: Position of the specimen selected for preservation in herbarium (left or right).
Column 7: The relative level of insect damage in the specimen selected for preservation in herbarium (low or high).

```
Observer01 Acer right 2.64 2.64 right low
Observer01 Acer2 left 5.67 4.46 left low
Observer01 Alnus left 0.32 0.18 left low
Observer01 Amellanchier right 14.21 13.84 left hig
Observer01 Betula left 5.33 5.28 left low
Observer01 Carpinus left 5.11 2.15 right hig
Observer01 Castanea left 16.62 16.68 left low
Observer01 Corylus right 8.29 8.26 right low
Observer01 Fagus left 4.78 4.24 left low
Observer01 Malus right 3.7 3.52 left hig
Observer01 Padus left 4.8 3.25 left low
Observer01 Populus right 0.22 0.16 right low
Observer01 Quercus right 4.42 2.87 right low
Observer01 Quercus2 left 16.88 6.59 right hig
Observer01 Rhamnus right 2.52 1.84 left hig
Observer01 Rhododendron right 0.65 0.49 right low
Observer01 Rubus right 0.72 0.66 right low
Observer01 Salix right 0.43 0.21 right low
Observer01 Sorbus left 1.75 1.75 right hig
Observer01 Tilia left 10.32 10.02 right hig
Observer01 Ulmus right 3.59 3.31 right low
Observer02 Acer right 2.64 2.64 right low
Observer02 Acer2 left 5.67 4.46 left low
Observer02 Alnus left 0.32 0.18 left low
Observer02 Amellanchier right 14.21 13.84 right low
Observer02 Betula left 5.33 5.28 left low
Observer02 Carpinus left 5.11 2.15 right hig
Observer02 Castanea left 16.62 16.68 left low
Observer02 Corylus right 8.29 8.26 right low
Observer02 Fagus left 4.78 4.24 left low
Observer02 Malus right 3.7 3.52 left hig
Observer02 Padus left 4.8 3.25 left low
Observer02 Populus right 0.22 0.16 right low
Observer02 Quercus right 4.42 2.87 right low
Observer02 Quercus2 left 16.88 6.59 left low
Observer02 Rhamnus right 2.52 1.84 left hig
Observer02 Rhododendron right 0.65 0.49 right low
Observer02 Rubus right 0.72 0.66 left hig
Observer02 Salix right 0.43 0.21 left hig
Observer02 Sorbus left 1.75 1.75 left low
Observer02 Tilia left 10.32 10.02 right hig
Observer02 Ulmus right 3.59 3.31 left hig
```

```
Observer03 Acer right 2.64 2.64 right low
Observer03 Acer2 left 5.67 4.46 left low
Observer03 Alnus left 0.32 0.18 left low
Observer03 Amellanchier right 14.21 13.84 right low
Observer03 Betula left 5.33 5.28 left low
Observer03 Carpinus left 5.11 2.15 left low
Observer03 Castanea left 16.62 16.68 left low
Observer03 Corylus right 8.29 8.26 right low
Observer03 Fagus left 4.78 4.24 left low
Observer03 Malus right 3.7 3.52 right low
Observer03 Padus left 4.8 3.25 left low
Observer03 Populus right 0.22 0.16 right low
Observer03 Quercus right 4.42 2.87 right low
Observer03 Quercus2 left 16.88 6.59 left low
Observer03 Rhamnus right 2.52 1.84 left hig
Observer03 Rhododendron right 0.65 0.49 right low
Observer03 Rubus right 0.72 0.66 right low
Observer03 Salix right 0.43 0.21 left hig
Observer03 Sorbus left 1.75 1.75 left low
Observer03 Tilia left 10.32 10.02 left low
Observer03 Ulmus right 3.59 3.31 right low
Observer04 Acer right 2.64 2.64 right low
Observer04 Acer2 left 5.67 4.46 left low
Observer04 Alnus left 0.32 0.18 left low
Observer04 Amellanchier right 14.21 13.84 right low
Observer04 Betula left 5.33 5.28 left low
Observer04 Carpinus left 5.11 2.15 right hig
Observer04 Castanea left 16.62 16.68 left low
Observer04 Corylus right 8.29 8.26 right low
Observer04 Fagus left 4.78 4.24 right hig
Observer04 Malus right 3.7 3.52 left hig
Observer04 Padus left 4.8 3.25 left low
Observer04 Populus right 0.22 0.16 right low
Observer04 Quercus right 4.42 2.87 right low
Observer04 Quercus2 left 16.88 6.59 left low
Observer04 Rhamnus right 2.52 1.84 left hig
Observer04 Rhododendron right 0.65 0.49 left hig
Observer04 Rubus right 0.72 0.66 right low
Observer04 Salix right 0.43 0.21 left hig
Observer04 Sorbus left 1.75 1.75 right hig
Observer04 Tilia left 10.32 10.02 right hig
Observer04 Ulmus right 3.59 3.31 right low
Observer05 Acer right 2.64 2.64 right low
Observer05 Acer2 left 5.67 4.46 left low
Observer05 Alnus left 0.32 0.18 left low
Observer05 Amellanchier right 14.21 13.84 right low
Observer05 Betula left 5.33 5.28 left low
Observer05 Carpinus left 5.11 2.15 right hig
Observer05 Castanea left 16.62 16.68 left low
Observer05 Corylus right 8.29 8.26 right low
Observer05 Fagus left 4.78 4.24 left low
Observer05 Malus right 3.7 3.52 left hig
Observer05 Padus left 4.8 3.25 left low
Observer05 Populus right 0.22 0.16 right low
Observer05 Quercus right 4.42 2.87 right low
Observer05 Quercus2 left 16.88 6.59 left low
Observer05 Rhamnus right 2.52 1.84 left hig
Observer05 Rhododendron right 0.65 0.49 left hig
Observer05 Rubus right 0.72 0.66 left hig
Observer05 Salix right 0.43 0.21 left hig
Observer05 Sorbus left 1.75 1.75 left low
```

```
Observer05 Tilia left 10.32 10.02 left low
Observer05 Ulmus right 3.59 3.31 right low
Observer06 Acer right 2.64 2.64 right low
Observer06 Acer2 left 5.67 4.46 left low
Observer06 Alnus left 0.32 0.18 left low
Observer06 Amellanchier right 14.21 13.84 right low
Observer06 Betula left 5.33 5.28 left low
Observer06 Carpinus left 5.11 2.15 left low
Observer06 Castanea left 16.62 16.68 left low
Observer06 Corylus right 8.29 8.26 right low
Observer06 Fagus left 4.78 4.24 left low
Observer06 Malus right 3.7 3.52 right low
Observer06 Padus left 4.8 3.25 left low
Observer06 Populus right 0.22 0.16 right low
Observer06 Quercus right 4.42 2.87 right low
Observer06 Quercus2 left 16.88 6.59 right hig
Observer06 Rhamnus right 2.52 1.84 left hig
Observer06 Rhododendron right 0.65 0.49 right low
Observer06 Rubus right 0.72 0.66 right low
Observer06 Salix right 0.43 0.21 left hig
Observer06 Sorbus left 1.75 1.75 right hig
Observer06 Tilia left 10.32 10.02 right hig
Observer06 Ulmus right 3.59 3.31 right low
Observer07 Acer right 2.64 2.64 right low
Observer07 Acer2 left 5.67 4.46 left low
Observer07 Alnus left 0.32 0.18 left low
Observer07 Amellanchier right 14.21 13.84 right low
Observer07 Betula left 5.33 5.28 left low
Observer07 Carpinus left 5.11 2.15 right hig
Observer07 Castanea left 16.62 16.68 right hig
Observer07 Corylus right 8.29 8.26 right low
Observer07 Fagus left 4.78 4.24 right hig
Observer07 Malus right 3.7 3.52 right low
Observer07 Padus left 4.8 3.25 left low
Observer07 Populus right 0.22 0.16 right low
Observer07 Quercus right 4.42 2.87 right low
Observer07 Quercus2 left 16.88 6.59 left low
Observer07 Rhamnus right 2.52 1.84 left hig
Observer07 Rhododendron right 0.65 0.49 left hig
Observer07 Rubus right 0.72 0.66 right low
Observer07 Salix right 0.43 0.21 right low
Observer07 Sorbus left 1.75 1.75 left low
Observer07 Tilia left 10.32 10.02 left low
Observer07 Ulmus right 3.59 3.31 right low
Observer08 Acer right 2.64 2.64 right low
Observer08 Acer2 left 5.67 4.46 left low
Observer08 Alnus left 0.32 0.18 left low
Observer08 Amellanchier right 14.21 13.84 right low
Observer08 Betula left 5.33 5.28 left low
Observer08 Carpinus left 5.11 2.15 right hig
Observer08 Castanea left 16.62 16.68 right hig
Observer08 Corylus right 8.29 8.26 right low
Observer08 Fagus left 4.78 4.24 right hig
Observer08 Malus right 3.7 3.52 right low
Observer08 Padus left 4.8 3.25 left low
Observer08 Populus right 0.22 0.16 right low
Observer08 Quercus right 4.42 2.87 right low
Observer08 Quercus2 left 16.88 6.59 left low
Observer08 Rhamnus right 2.52 1.84 left hig
Observer08 Rhododendron right 0.65 0.49 left hig
Observer08 Rubus right 0.72 0.66 left hig
```

```
Observer08 Salix right 0.43 0.21 left hig
Observer08 Sorbus left 1.75 1.75 right hig
Observer08 Tilia left 10.32 10.02 left low
Observer08 Ulmus right 3.59 3.31 left hig
Observer09 Acer right 2.64 2.64 left hig
Observer09 Acer2 left 5.67 4.46 left low
Observer09 Alnus left 0.32 0.18 left low
Observer09 Amellanchier right 14.21 13.84 right low
Observer09 Betula left 5.33 5.28 left low
Observer09 Carpinus left 5.11 2.15 right hig
Observer09 Castanea left 16.62 16.68 right hig
Observer09 Corylus right 8.29 8.26 right low
Observer09 Fagus left 4.78 4.24 right hig
Observer09 Malus right 3.7 3.52 left hig
Observer09 Padus left 4.8 3.25 left low
Observer09 Populus right 0.22 0.16 right low
Observer09 Quercus right 4.42 2.87 right low
Observer09 Quercus2 left 16.88 6.59 left low
Observer09 Rhamnus right 2.52 1.84 left hig
Observer09 Rhododendron right 0.65 0.49 left hig
Observer09 Rubus right 0.72 0.66 left hig
Observer09 Salix right 0.43 0.21 right low
Observer09 Sorbus left 1.75 1.75 right hig
Observer09 Tilia left 10.32 10.02 left low
Observer09 Ulmus right 3.59 3.31 right low
Observer10 Acer right 2.64 2.64 right low
Observer10 Acer2 left 5.67 4.46 left low
Observer10 Alnus left 0.32 0.18 left low
Observer10 Amellanchier right 14.21 13.84 right low
Observer10 Betula left 5.33 5.28 left low
Observer10 Carpinus left 5.11 2.15 right hig
Observer10 Castanea left 16.62 16.68 left low
Observer10 Corylus right 8.29 8.26 right low
Observer10 Fagus left 4.78 4.24 right hig
Observer10 Malus right 3.7 3.52 right low
Observer10 Padus left 4.8 3.25 left low
Observer10 Populus right 0.22 0.16 right low
Observer10 Quercus right 4.42 2.87 right low
Observer10 Quercus2 left 16.88 6.59 left low
Observer10 Rhamnus right 2.52 1.84 left hig
Observer10 Rhododendron right 0.65 0.49 left hig
Observer10 Rubus right 0.72 0.66 right low
Observer10 Salix right 0.43 0.21 left hig
Observer10 Sorbus left 1.75 1.75 right hig
Observer10 Tilia left 10.32 10.02 left low
Observer10 Ulmus right 3.59 3.31 right low
Observer11 Acer right 2.64 2.64 right low
Observer11 Acer2 left 5.67 4.46 left low
Observer11 Alnus left 0.32 0.18 left low
Observer11 Amellanchier right 14.21 13.84 right low
Observer11 Betula left 5.33 5.28 left low
Observer11 Carpinus left 5.11 2.15 right hig
Observer11 Castanea left 16.62 16.68 right hig
Observer11 Corylus right 8.29 8.26 right low
Observer11 Fagus left 4.78 4.24 left low
Observer11 Malus right 3.7 3.52 right low
Observer11 Padus left 4.8 3.25 left low
Observer11 Populus right 0.22 0.16 right low
Observer11 Quercus right 4.42 2.87 right low
Observer11 Quercus2 left 16.88 6.59 left low
Observer11 Rhamnus right 2.52 1.84 left hig
```

```
Observer11 Rhododendron right 0.65 0.49 right low
Observer11 Rubus right 0.72 0.66 right low
Observer11 Salix right 0.43 0.21 right low
Observer11 Sorbus left 1.75 1.75 left low
Observer11 Tilia left 10.32 10.02 left low
Observer11 Ulmus right 3.59 3.31 right low
Observer12 Acer right 2.64 2.64 left hig
Observer12 Acer2 left 5.67 4.46 right hig
Observer12 Alnus left 0.32 0.18 left low
Observer12 Amellanchier right 14.21 13.84 left hig
Observer12 Betula left 5.33 5.28 left low
Observer12 Carpinus left 5.11 2.15 right hig
Observer12 Castanea left 16.62 16.68 right hig
Observer12 Corylus right 8.29 8.26 right low
Observer12 Fagus left 4.78 4.24 right hig
Observer12 Malus right 3.7 3.52 left hig
Observer12 Padus left 4.8 3.25 left low
Observer12 Populus right 0.22 0.16 right low
Observer12 Quercus right 4.42 2.87 right low
Observer12 Quercus2 left 16.88 6.59 left low
Observer12 Rhamnus right 2.52 1.84 left hig
Observer12 Rhododendron right 0.65 0.49 left hig
Observer12 Rubus right 0.72 0.66 left hig
Observer12 Salix right 0.43 0.21 left hig
Observer12 Sorbus left 1.75 1.75 right hig
Observer12 Tilia left 10.32 10.02 right hig
Observer12 Ulmus right 3.59 3.31 right low
Observer13 Acer right 2.64 2.64 left hig
Observer13 Acer2 left 5.67 4.46 left low
Observer13 Alnus left 0.32 0.18 left low
Observer13 Amellanchier right 14.21 13.84 left hig
Observer13 Betula left 5.33 5.28 left low
Observer13 Carpinus left 5.11 2.15 right hig
Observer13 Castanea left 16.62 16.68 right hig
Observer13 Corylus right 8.29 8.26 right low
Observer13 Fagus left 4.78 4.24 left low
Observer13 Malus right 3.7 3.52 right low
Observer13 Padus left 4.8 3.25 left low
Observer13 Populus right 0.22 0.16 left hig
Observer13 Quercus right 4.42 2.87 left hig
Observer13 Quercus2 left 16.88 6.59 left low
Observer13 Rhamnus right 2.52 1.84 left hig
Observer13 Rhododendron right 0.65 0.49 left hig
Observer13 Rubus right 0.72 0.66 left hig
Observer13 Salix right 0.43 0.21 left hig
Observer13 Sorbus left 1.75 1.75 right hig
Observer13 Tilia left 10.32 10.02 right hig
Observer13 Ulmus right 3.59 3.31 right low
Observer14 Acer right 2.64 2.64 right low
Observer14 Acer2 left 5.67 4.46 left low
Observer14 Alnus left 0.32 0.18 left low
Observer14 Amellanchier right 14.21 13.84 right low
Observer14 Betula left 5.33 5.28 left low
Observer14 Carpinus left 5.11 2.15 right hig
Observer14 Castanea left 16.62 16.68 left low
Observer14 Corylus right 8.29 8.26 right low
Observer14 Fagus left 4.78 4.24 left low
Observer14 Malus right 3.7 3.52 right low
Observer14 Padus left 4.8 3.25 left low
Observer14 Populus right 0.22 0.16 right low
Observer14 Quercus right 4.42 2.87 right low
```

```
Observer14 Quercus2 left 16.88 6.59 left low
Observer14 Rhamnus right 2.52 1.84 left hig
Observer14 Rhododendron right 0.65 0.49 left hig
Observer14 Rubus right 0.72 0.66 left hig
Observer14 Salix right 0.43 0.21 right low
Observer14 Sorbus left 1.75 1.75 right hig
Observer14 Tilia left 10.32 10.02 left low
Observer14 Ulmus right 3.59 3.31 right low
Observer15 Acer right 2.64 2.64 right low
Observer15 Acer2 left 5.67 4.46 left low
Observer15 Alnus left 0.32 0.18 left low
Observer15 Amellanchier right 14.21 13.84 right low
Observer15 Betula left 5.33 5.28 left low
Observer15 Carpinus left 5.11 2.15 right hig
Observer15 Castanea left 16.62 16.68 right hig
Observer15 Corylus right 8.29 8.26 right low
Observer15 Fagus left 4.78 4.24 left low
Observer15 Malus right 3.7 3.52 right low
Observer15 Padus left 4.8 3.25 left low
Observer15 Populus right 0.22 0.16 right low
Observer15 Quercus right 4.42 2.87 left hig
Observer15 Quercus2 left 16.88 6.59 left low
Observer15 Rhamnus right 2.52 1.84 left hig
Observer15 Rhododendron right 0.65 0.49 left hig
Observer15 Rubus right 0.72 0.66 right low
Observer15 Salix right 0.43 0.21 left hig
Observer15 Sorbus left 1.75 1.75 left low
Observer15 Tilia left 10.32 10.02 right hig
Observer15 Ulmus right 3.59 3.31 right low
```
